# The Application of Green Solvents in the Synthesis of *S*-Heterocyclic Compounds—A Review

**DOI:** 10.3390/ijms25179474

**Published:** 2024-08-31

**Authors:** Tomasz Kosmalski, Renata Kołodziejska, Monika Przybysz, Łukasz Szeleszczuk, Hanna Pawluk, Katarzyna Mądra-Gackowska, Renata Studzińska

**Affiliations:** 1 Department of Organic Chemistry, Faculty of Pharmacy, Collegium Medicum in Bydgoszcz, Nicolaus Copernicus University in Toruń, 2 Jurasza Str., 85-089 Bydgoszcz, Poland; tkosm@cm.umk.pl (T.K.); monika.przybysz@cm.umk.pl (M.P.); 2 Department of Medical Biology and Biochemistry, Faculty of Medicine, Collegium Medicum in Bydgoszcz, Nicolaus Copernicus University in Toruń, 24 Karłowicza Str., 85-092 Bydgoszcz, Poland; renatak@cm.umk.pl (R.K.); hannapawluk@cm.umk.pl (H.P.); 3 Department of Organic and Physical Chemistry, Faculty of Pharmacy, Medical University of Warsaw, 1 Banacha Str., 02-093 Warsaw, Poland; lukasz.szeleszczuk@wum.edu.pl; 4 Department of Geriatrics, Faculty of Health Sciences, Collegium Medicum in Bydgoszcz, Nicolaus Copernicus University in Toruń, 9 Skłodowskiej Curie Str., 85-094 Bydgoszcz, Poland; katarzyna.madra@cm.umk.pl

**Keywords:** *S*-heterocycles, green synthesis, green solvents, ionic liquids

## Abstract

Cyclic organic compounds containing sulfur atoms constitute a large group, and they play an important role in the chemistry of heterocyclic compounds. They are valuable intermediates for the synthesis of other compounds or biologically active compounds themselves. The synthesis of heterocyclic compounds poses a major challenge for organic chemists, especially in the context of applying the principles of “green chemistry”. This work is a review of the methods of synthesis of various *S*-heterocyclic compounds using green solvents such as water, ionic liquids, deep eutectic solvents, glycerol, ethylene glycol, polyethylene glycol, and sabinene. The syntheses of five-, six-, and seven-membered heterocyclic compounds containing a sulfur atom or atoms, as well as those with other heteroatoms and fused-ring systems, are described. It is shown that using green solvents determines the attractiveness of conditions for many reactions; for others, such use constitutes a real compromise between efficiency and mild reaction conditions.

## 1. Introduction

Heterocyclic compounds are a wide range of structures with one or more rings in which heteroatoms are present (usually nitrogen, oxygen, and sulfur). The number of possible heterocyclic systems is practically unlimited. They are widely used as pesticides, insecticides, herbicides, and rodenticides. Further important practical applications include dyestuffs, copolymers, solvents, photographic sensitizers and developers, and antioxidants and vulcanization accelerators in the rubber industry. Many heterocycles are valuable intermediates in synthesis [1]. 

Heterocyclic compounds are prevalent and necessary for life, playing an important role in the metabolism of all living cells. Examples can include pyrimidine and purine bases, which are components of nucleic acids or some amino acids [1].

**Figure 1:**
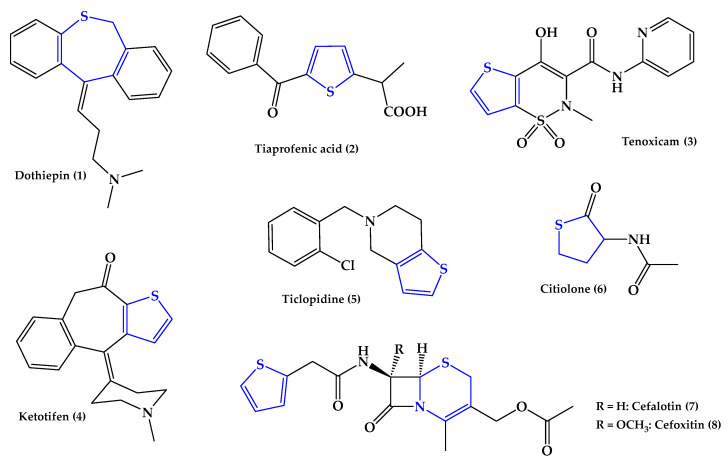
Examples of structures of drugs containing heterocyclic groups with sulfur atoms. Heterocyclic fragments of molecules containing sulfur are marked in blue.

Heterocyclic systems containing various heteroatoms are also structural components of many pharmacologically active compounds, both natural and synthetic. Of these, the compounds containing sulfur atoms, both as the only heteroatom and as one of several heteroatoms, deserve attention. Examples of structures of drugs containing heterocyclic groups with sulfur atoms are shown in Figure 1.

Dothiepin (**1**) is a tricyclic antidepressant that is a structural analog of amitriptyline. It is used in medium-intensified depressive syndromes of different causes that occur with fear and anxiety5[2]. It has an antidepressant effect weaker than that of amitriptyline but stronger anxiolytic and sedative effects.

Tiaprofenic (**2**) acid is a known example of a commercially available drug with anti-inflammatory properties that contains a thiophene ring as a pharmacophoric group. It is a non-steroidal anti-inflammatory drug (NSAID), the derivative of propionic acid used in the treatment of pain and inflammation. It acts by inhibiting COX enzymes [3].

Tenoxicam (TNX) (**3**) is also a non-steroidal anti-inflammatory medication. It belongs to the oxicam family. TNX is a long-acting drug with powerful, antipyretic, anti-inflammatory, and analgesic effects. Consequently, TNX effectively treats many inflammatory diseases involving musculoskeletal and joint diseases such as osteoarthritis, gout, rheumatoid arthritis, backache, ankylosing spondylitis, bursitis, tendonitis, etc. [4].

Ketotifen (**4**) is a derivative of a tri-cyclic ring system of benzocycloheptatiophen. It is an antihistamine drug used to treat allergic diseases, and it is used as an anti-asthma agent. It shows the ability to inhibit histamine release from the mast cells and basophils. It causes the long-term braking of histamine reactions by blocking H_1_ receptors [5].

Ticlopidine (**5**) is a derivative of thiophene pyridine that has an inhibitory effect on platelet aggregation via various inducers, such as adenosine diphosphate (ADP) (including exogenous and endogenous ADP), collagen, thrombin, arachidonic acid, and peroxides in prostaglandins. It is commonly used in patients with coronary heart disease, cerebral infarction, a thrombus, occlusive arteriosclerosis, arterial obliterans, etc. Ticlopidine is used in peripheral arterial and brain-vessel thrombosis [6].

Citiolone (**6**) is a thiol-derived drug that is often used as a mucolytic agent for the treatment of certain liver diseases and to protect liver-cell membranes. It can also be used clinically for the treatment of infectious and toxic hepatitis [7].

Cefalotin (**7**) is an antibiotic belonging to the first, oldest generation of cephalosporin, i.e., β-lactam antibiotics, whose structure and mechanism of action are similar to those of penicillins. Cefoxitin (**8**), in turn, is a beta-lactam antibiotic belonging to the second generation of cephalosporin. Its resistance to destruction via beta-lactamases results in a broad spectrum of antibacterial activity, which includes anaerobic and Gram-positive and Gram-negative aerobic bacteria, including many resistant to cephalothin and other cephalosporins [8].

The presence of heterocyclic systems containing sulfur in many biologically active compounds, as well as the possibility of using this type of compound in many other areas, generates the need for a synthesis of new substances containing this type of structural fragment.

To avoid the excessive use of solvents that are unfavorable to the environment, the synthesis of this type is increasingly conducted using the principles of “green chemistry”.

## 2. Solvents in Organic Synthesis

Solvents are used daily in many industrial processes as reaction media, in separation procedures, and as diluters. Most often, they are not an integral part of the compounds undergoing a reaction, but they play an important role in chemical production and synthesis [9]. On the one hand, the ability to use a wide variety of organic solvents has resulted in significant advances in chemical synthesis, but on the other hand, the use of solvents causes many environmental and health problems that have been observed since the 1940s [10].

Large amounts of solvents are required to carry out chemical reactions concerning the reagents used. In addition, many of the processes necessary to obtain high-purity products also require the use of large excess amounts. The annual production of organic solvents on an industrial scale has been estimated at 200 million tons [11].

The excessive use of non-renewable, toxic solvents is harmful to the environment, and it is a perfect example of unsustainable practices. On the other hand, solvents are essential in many chemical processes [12]. 

Usually, a process solvent is a liquid, non-reactive auxiliary agent that dissolves reactants and facilitates the separation of products via crystallization or chromatography. The solvent plays an integral role in reaction mixtures. Intermolecular interactions with the solvent stabilize the solutes, which can facilitate the desired equilibrium position, favorably adjust the kinetic profile of the reaction, and also affect product selectivity [13].

A significant source of chemical waste is the small-scale chemical and pharmaceutical industries. This is mainly due to the production of complex molecules that involve large amounts of solvents during synthesis, as well as purification [14].

Solvents are often the main component in process chemistry, especially when we need to build molecular complexity. It requires multiple steps with a lot of various solvents. The treatment procedure after each reaction increases the need for a solvent. A typical example of this type of production is active pharmaceutical components’ synthesis (APIs) [14].

A study by the ACS Green Chemical Institute (GCI) found that 56% of the materials used in pharmaceutical production were solvents, with water accounting for another 32% [15].

However, in terms of quantity, large-scale sectors, i.e., the oil-refining industry and bulk chemical industry, produce more waste than the small-scale chemical and pharmaceutical sectors [12].

The U.S. Environmental Protection Agency (EPA) launched the “Alternative Synthetic Routes for Pollution Prevention” program, which has proposed green chemistry for new technological solutions; the main concept is to reduce the amount of toxic, unwanted waste that negatively affects the environment. The program includes innovative approaches in many areas of chemistry related to organic synthesis, as well as in analytical chemistry, by eliminating or reducing the amount of reagents and solvents [10].

The economic and environmental need to minimize the use of solvents depends largely on the practicality of the recycling process and reuse. The reusability of a solvent can be of great benefit due to the volumes in which it is used [16]. Despite chemical diversity, a common feature of most solvents is volatility. On the one hand, this property facilitates the reuse of solvents due to possible distillation. On the other hand, it means a higher rate of evaporation, contributing to harmful emissions into the atmosphere.

Due to the intensification of regulations and regulations increasingly limiting the use of certain solvents (including volatile organic compounds with a low boiling point) and, at the same time, the necessary use of solvents for many chemical processes, other options are proposed as so-called “green alternatives”. With the arrival of green chemistry (and especially research on ionic liquids), the number of chemicals considered solvents has increased significantly, representing new possibilities in solvent applications. There has been a lot of interest in so-called “green” solvents, which are more environmentally friendly and can be equally or more efficient to use in comparison to conventional organic solvents. Since the beginning of 2000, it has been possible to observe a significant increase in the amount of research being carried out on the use of known solvents with the right parameters to meet the requirements of green chemistry and the design of new “green” solvents [17]. Examples of “green” solvents include water, ionic liquids (ILs), glycerol, supercritical carbon dioxide (CO_2_), deep eutectic solvents (DESs), natural deep eutectic solvents (NADESs), liquid polymers such as polyethylene glycol (PEG), and vegetable oils [12,18].

## 3. Synthesis of *S*-Heterocycles in Water as a Green Solvent

### 3.1. Synthesis of S-Heterocycles in Water Using Conventional Methods

Water has many advantages in comparison to conventional organic solvents. It has unique physicochemical properties, i.e., the formation of hydrogen bonds, a high heat capacity, a high dielectric constant, and a large temperature window, i.e., an area with the optimal temperature for a reaction. It is classified as a green solvent due to its cost, easy availability, non-toxicity, non-volatility, and non-flammability. Despite the many advantages of water, it is not commonly used as the sole solvent in chemical synthesis because most organic compounds are not water-soluble [18]. Scientists did not use water as a solvent due to the old doctrine that insoluble reagents do not have any effect. Over time, however, it has been taken into account that reactions can proceed “in” or “on” water, meaning that solubility is not important to these reactions [19]. In “on-water” reactions, water-insoluble reactants react by floating on water to produce a water-insoluble and easily separable final product [20]. An essential physical requirement for the on-water reactions of water-insoluble organic reactants is the presence of a liquid, organic, oily phase that interfaces with the bulk water layer. The term “on-water reaction” applies to organic reactions that occur between water-insoluble reactants at the interface of a bulk liquid-water phase that contains no additives. It does not apply to reactions in the presence of very small quantities of water, such as hydrated salts, or those involving other catalysts. If the organic reactants are solids, at least one must be liquefied to produce this organic oily layer [21]. Many organic syntheses have been carried in water. Some reactions take place with or without organometallic catalysts. Water, as a solvent, is also used in reactions assisted via microwave radiation or ultrasound [22].

**Figure 2:**
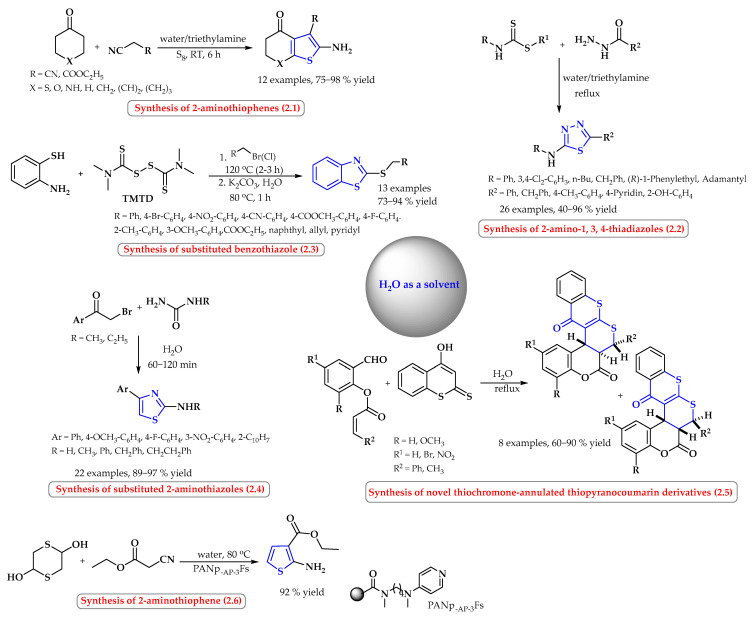
Synthesis of *S*-heterocyclic compounds in water as a solvent. Heterocyclic fragments of molecules containing sulfur are marked in blue.

Examples of reactions for the synthesis of sulfur heterocyclic compounds taking place in water without the use of a catalyst are reactions to obtain, among others, 2-aminotiophenes, 2-aminotiazoles, thiobenzoazoles, dithiocoumarins, and thiadiazoles (Figure 2).

Twelve different 2-aminotiophenes were obtained with good yields (75–98%) in a mixture of triethylamine and water (1:1 *v*/*v*) at room temperature (Figure 2, reaction 2.1). The Gewald reaction of α-methylene ketones and nitriles with elemental sulfur led to the desired products being obtained. Due to the high polarization of the medium, the products precipitate spontaneously in the reaction mixtures, which facilitates the process of their isolation [23]. Similarly, 2-amino-1,3,4-thiadiazoles were synthesized from the appropriate hydrazides and dithiocarbamates in an aqueous system with triethylamine (Figure 2, reaction 2.2). The process was carried out at the boiling point [24].

The synthesis of 2-benzyl substituted benzothiazoles starting from 2-aminothiophenols can be performed using tetramethyliuram disulfide (TMTD) under transition-metal-free conditions using water as the solvent (Figure 2, reaction 2.3). Transiently formed mercapto heterocycles and the subsequent C-S coupling with benzyl or allyl halides furnished the final products. The optimal reagent ratio for 2-aminothiophenol/TMTD/halide was 1:0.6:1 when potassium carbonate was used as the base. The authors presented 13 final products; the yields of the products were high (73–94%) [25].

However, 2-aminothiazoles were synthesized in water alone without a co-solvent at an ambient temperature (Figure 2, reaction 2.4). The developed protocol allowed the desired products to be obtained in a short time (up to 2 h) with very good efficiency of over 89% [26].

Moghaddam et al. reported the efficient synthesis of pentacyclic thiochromone derivatives via the Knoevenagel–hetero-Diels–Alder domino reaction in the absence of catalysts (Figure 2, reaction 2.5). With water used as a solvent, 4-hydroxydithiocoumarins and *O*-acrylated salicylaldehyde derivatives were heated to obtain reaction products with good yields and high regio- and stereoselectivity [27].

In 2015, Li and co-workers reported a synthetic pathway for the synthesis of 2-aminothiophene in water using 4-dimethylaminopyridine-functionalized polyacrylonitrile fiber as a catalyst (Figure 2, reaction 2.6) [28]. The product was obtained with a 92% yield in the reaction of 2,5-dihydroxy-1,4-dithiane with ethyl cyanoacetate. The reaction was carried out at 80 °C.

### 3.2. Synthesis of S-Heterocycles in Water Using Microwave or Ultrasound Radiation

In recent years, the use of radiation of various wavelengths, i.e., microwave radiation or ultrasound, in organic synthesis has become increasingly popular. Ultrasonic radiation increases the rate of many chemical reactions [22]. Ultrasonic waves in the liquid phase cause cavitation, including bubble nucleation, growth, and collapse. Bubble collapse occurs at millions of locations in the liquid medium, causing supercritical conditions such as a high temperature (5000 K) and high pressure (1000 atm) [29,30,31]. Through the sonic ionization of water due to cavitation, mechanical mixing, reaction pressures, and temperatures, the concentrations of hydrogen ions are increased. Additionally, water transmits ultrasonic energy more efficiently than other solvents. As a result, as the ultrasonic power increases, a lot of energy is supplied to the reaction mixture to enhance the cavitation effect.

Generally, ultrasonic-assisted reactions are more efficient and more selective, and they often do not require the use of chemical catalysts [32]. 

**Figure 3:**
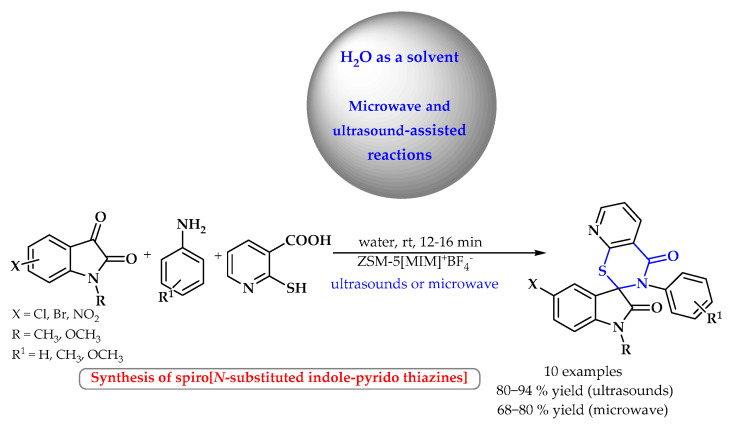
Microwave- or ultrasound-assisted synthesis of *S*-heterocyclic compounds in water as a solvent. Heterocyclic fragments of molecules containing sulfur are marked in blue.

*N*-substituted spiro[indole-pyrido[3,2-*e*]-thiazine] derivatives were synthesized by Kapil’s group (Figure 3) [33]. In a one-step reaction from the appropriate amines, 2-mercaptonicotinic acid and *N*-substituted indole 2,3-dione, in the presence of the ZSM-5-([MIM^+^]BF_4_^−^) catalyst under aqueous conditions and under the influence of ultrasounds, ten different thiazine derivatives were obtained using ultrasonic radiation with high efficiency (80–94%). Lower yields (68–80%) were obtained if the reaction was carried out at a similar time under the influence of microwave radiation.

**Figure 4:**
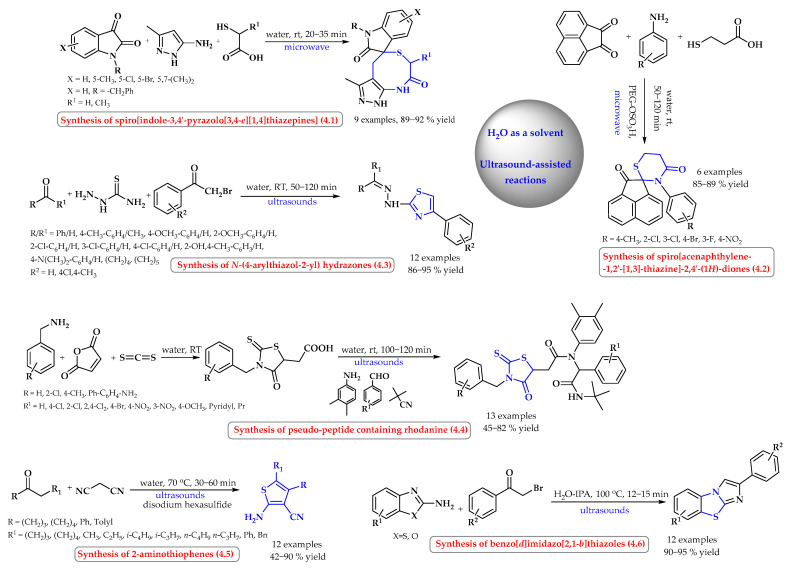
Ultrasound-assisted synthesis of *S*-heterocyclic compounds in water as a solvent. Heterocyclic fragments of molecules containing sulfur are marked in blue.

A series of spiroindole-thiazepines and thiazolidines were obtained in water at room temperature under the influence of ultrasonic radiation in a one-step synthesis (Figure 4, reaction 4.1). In a domino reaction using 2-mercaptoacetic acid, isatin, and 3-amino-5-methylpyrazole, nine substituted spiro[indole-3,4′-pyrazolo[3,4-*e*][1,4]thiazepine derivatives were obtained with excellent yields of 89–92% (Figure 4) [34]. 

Similarly, in a one-pot procedure, six spiro[acenaphthylene-thiazine]diones were obtained from a three-component mixture of various anilines, 3-mercaptopropionic acid, and acenaphthalene-1,2-dione (Figure 4, reaction 4.2). In the presence of a polymer-supported catalyst (PEG–OSO_3_H) in an aqueous medium and at room temperature, irradiation yields spirotiazines with an 85–89% yield [35]. 

However, Zhang et al. reported a simple one-step reaction for twelve 2-benzylidenehydrazo-4-phenylthiazole derivatives (Figure 4, reaction 4.3) [36]. The reaction between various carbonyl compounds, thiosemicarbazide, and phenacyl bromide in an aqueous solvent via ultrasonic irradiation at room temperature provided an 86–95% product. All twelve reactions were completed in a shorter time and with better efficiency in the ultrasound experiment compared to the conventional method (50–120 min versus 90–240 min; efficiency 86–95% versus 60–89%).

Five-membered rhodanine derivatives containing a thiazolidine unit were also synthesized in a sequential reaction in water under ultrasonic radiation at room temperature (Figure 4, reaction 4.4). Thirteen different derivatives were obtained in yields of 45–82% [37].

The synthesis of 2-aminotiophenes was also carried out in water after ultrasonic activation (Figure 4, reaction 4.5). In the reaction of appropriate ketones with malononitrile in the presence of sodium polysulfides at a temperature of 70 °C, condensation products were obtained with a 42–90% yield [38].

The ultrasound-assisted reaction between substituted 2-aminobenzothiazoles and phenacyl bromides with both electron-donating and electron-withdrawing substituents produced the corresponding benzo[*d*]imidazo[2,1-*b*]thiazoles in yields above 90% (Figure 4, reaction 4.6). The process was carried out at a temperature of 100 °C in water with the addition of isopropyl alcohol (IPA) as a co-solvent to increase the solubility of the substrates [39].

## 4. Synthesis of *S*-Heterocycles in Ionic Liquids as Green Solvents

**Figure 5:**
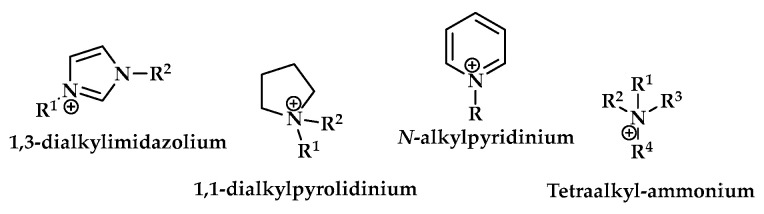
The organic cations most commonly used in ionic liquids.

Ionic liquids (ILs) are organic salts made up of various organic cations and inorganic or organic anions. The most common cations are 1,3-dialkylimidazolium, 1,1-dialkylpyrrolidine, *N*-alkylpyridinium, or quaternary ammonium salt (Figure 5). Compared to standard organic solvents, ionic liquids have a low melting point, so most representatives of this class exist in a liquid state at room temperature.

By selecting the appropriate cation and anion and modifying the organic part (e.g., by introducing additional substituents), it is possible to design an ionic liquid with unique physical properties tailored to the specific requirements of the reaction. ILs are a green alternative to conventional organic solvents, with great potential due to outstanding chemical and thermal stability, high ionic and electrochemical conductivity, low vapor pressure, and the ability to interact with organic and inorganic substances. They are widely used in organic and inorganic synthesis, enzymatic and chemical catalysis, and biotransformation using whole microbial cells [17,18,40].

The physical, biological, and chemical properties of ionic liquids depend on the structure of the cation, its symmetry, the presence of alkyl groups with different hydrophobicity, and the degree of delocalization of the anion’s charge. A characteristic feature of ILs is a low melting point, ranging from 0 to 100 °C. Most ionic liquids are liquid at room temperature (RILs) and remain liquid over a wide temperature window below 300 °C. An increase in the melting point is observed with the elongation of the alkyl chain of the cation. On the other hand, the decrease in temperature is due to weaker molecular interactions (van der Waals forces, hydrogen bonds, and electrostatic interactions), lower structural symmetry, and evenly distributed ion charges. Most often, the decrease in the melting point is related to the size of the inorganic anion; a larger-volume anion endowed with the same charge contributes to the reduction in temperature [17,41,42,43,44,45].

Compared to conventional organic solvents, ionic liquids are much more viscous. For commonly used ILs, the viscosity ranges from 35 to 500 cP (for comparison: toluene—0.6 cP, methanol—0.5 cP, and water—0.9 cP). The viscosity of ionic liquids depends on the chemical structure, composition, intermolecular forces, the aliphatic chain length of substituents modifying the organic cation, the temperature, and the presence of co-solvents. The change in the viscosity of ILs is correlated with polarity and can be achieved by manipulating the length of the alkyl chain. Ionic liquids with shorter aliphatic chains have lower viscosity because they are more polar, while higher viscosity is exhibited by ILs with larger associated anion sizes.

The density of ILs ranges from 1.1 to 1.6 g·cm^−3^ and is generally higher than the density of conventional organic solvents or water. The density of an ionic liquid, like the viscosity, depends on the length and type of substituents in the cation, as well as the type of anion. In many cases, density decreases with a decreasing anion or increasing alkyl chain length of the cation. Replacing the hydrogen atom with heavier elements such as F, Cl, and Br also increases the density of ionic liquids. In addition, the density of ILs decreases linearly as the temperature increases [41,42,43,44,45].

Due to the diversity of ionic liquids in terms of their composition and properties, as well as the ability to “build” new ones, they are widely used in a variety of organic syntheses. Many cyclization reactions, leading to the formation of heterocyclic compounds, are carried out in ionic liquids.

**Figure 6:**
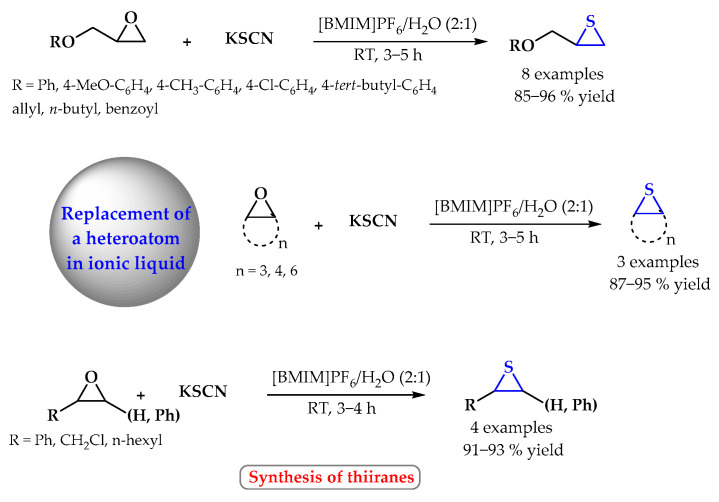
The conversion of oxiranes to thiiranes using ionic liquids. Heterocyclic fragments of molecules containing sulfur are marked in blue.

The conversion of oxiranes to thiiranes using ionic liquids is a convenient way to obtain heterocyclic derivatives with an incorporated sulfur atom. A variety of epoxides respond rapidly with potassium thiocyanate in a 1-butyl-3-methylimidazolium hexafluorophosphate [BMIM]PF_6_-H_2_O (2:1) solvent system at room temperature under mild and convenient conditions to produce the corresponding thiiranes in high to quantitative yields (Figure 6). The use of ionic liquids allows the use of heavy metal halides as promoters and chlorinated organic solvents to be avoided. Moreover, the ionic liquid can be recycled [46].

**Figure 7:**
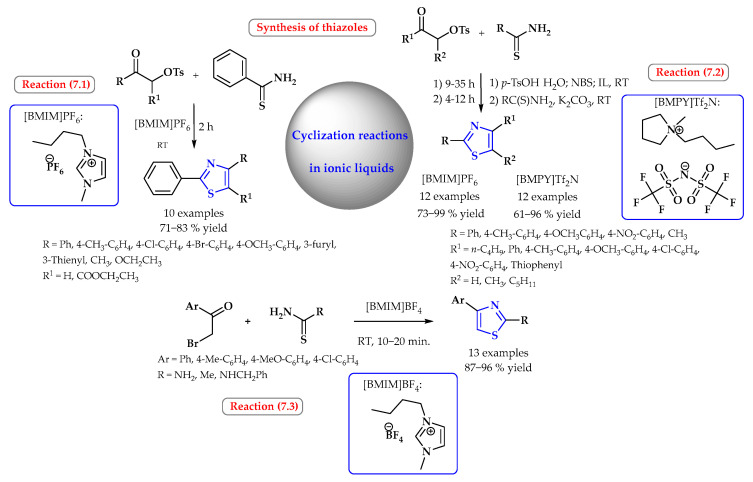
Synthesis of thiazoles in ionic liquids. Heterocyclic fragments of molecules containing sulfur are marked in blue.

The thiazoles were formed via a one-pot synthetic conversion of the appropriate ketones and thioamide through cyclocondensation using ionic liquids as the reaction medium (Figure 7). Reactions in ionic liquids were more efficient, taking place in a shorter time than in typical organic solvents, with the possibility of reuse without much of a decrease in the yield. In [BMIM]PF_6_, substituted 2-phenylthiazoles were synthesized via the cyclocondensation of α-tosyloxyketones with thiobenzamide at a yield of up to 83% (Figure 7, reaction 7.1) [47]. This method used an ionic liquid in a molar ratio of 1:10 (reagent/IL); the reactions were carried out for two hours at room temperature. In comparison, obtaining one of the products 2,4-diphenylthiazole in the classical molecular solvents, such as dichloromethane, required refluxing for 7 h [47].

The one-pot conversion of ketones into thiazoles through treatment with NBS, and subsequently with thioamides, could also be carried out in [BMIM]PF_6_ and *N*-butyl-*N*-methylpyrrolidinium bis(trifluoromethanesulfonyl)imidate ([BMPY]Tf_2_N) (Figure 7, reaction 7.2). The efficiency of the process in ionic liquids was, in most cases, higher than in chloroform [48].

Potewar et al. tested various [BMIM]X-type ionic liquids for the synthesis of substituted thiazoles. For the model reaction of phenacyl bromides and thiourea taking place at room temperature, the best yield was obtained in the ionic liquid 1-butyl-3-methylimidazolium tetrafluoroborate [BMIM]BF_4_ (96%) and the lowest for 1-butyl-3-methylimidazolium perchlorate [BMIM]ClO_4_ (48%). According to the authors, this is related to the increasing basicity of the anion; the p*K*a for [BMIM]BF_4_ is 0.5, and that for [BMIM]ClO_4_ is -11. In the ionic liquid [BMIM]BF_4_, a reaction with other substituted phenacyl bromides and thiourea and thioacetamides was carried out (Figure 7, reaction 7.3). The reaction time was 10–20 min, and the yields were very high, ranging from 87 to 96% [49]. 

**Figure 8:**
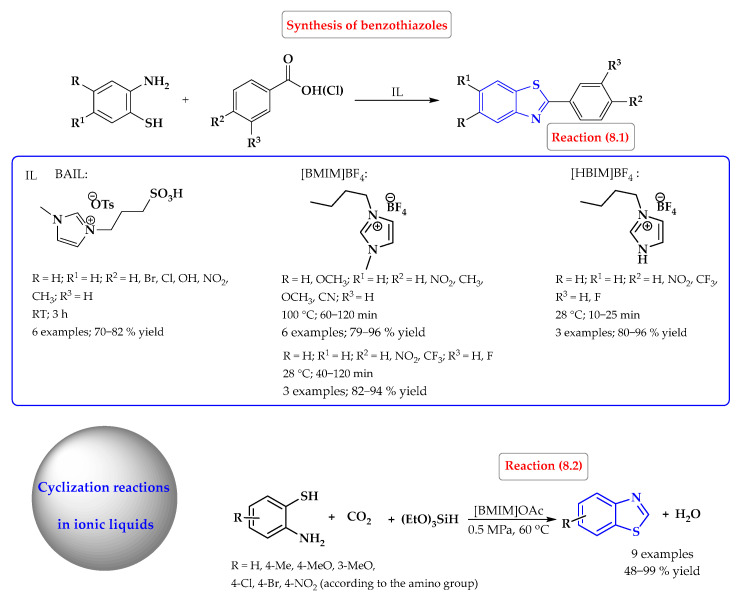
Synthesis of benzothiazoles in ionic liquids. Heterocyclic fragments of molecules containing sulfur are marked in blue.

A one-pot regioselective synthesis of 2-aryl benzothiazoles in excellent isolated yields has been developed using room-temperature ionic liquids as reaction media and promoters (Figure 8, reaction 8.1). 1-Butylimidazole tetrafluoroborate ([HBIM]BF_4_), [BMIM]BF_4_, and Brønsted acidic ionic liquid (BAIL) were used as ionic liquids. There was no need for the use of an additional catalyst normally employed in the methodologies reported so far. Benzotriazoles were produced by condensing a wide range of carboxylic acids or acid chlorides with 2-aminothiophenols [50,51,52].

However, in the work of Gao et al., the first protocol for the synthesis of benzothiazoles using CO_2_ as a C1 building block under metal-free and mild conditions (0.5 MPa, 60 °C) was developed using ionic liquids. After the process was optimized, the best reaction medium was selected: 1-butyl-3-methylimidazolium acetate ([BMIM][OAc]). A series of nine benzothiazoles were obtained from substituted 2-aminobenzenethiols, CO_2_, and triethoxysilane with yields ranging from 48% (with the 4-NO_2_ group) to 99% (for the unsubstituted system) (Figure 8, reaction 8.2) [53].

**Figure 9:**
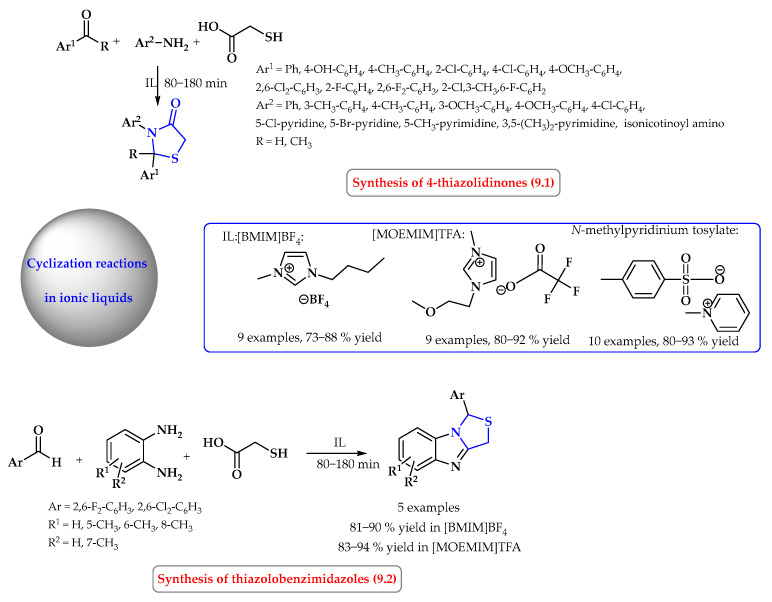
Synthesis of thiazolidine derivatives in ionic liquid. Heterocyclic fragments of molecules containing sulfur are marked in blue.

The synthesis of thiazolidinones from aromatic/heteryl amines, mercaptoacetic acid, and carbonyl compounds was carried out in various ionic liquids (Figure 9). The use of [BMIM]BF_4_ as the reaction medium allowed 2,3-disubstituted thiazolidinone derivatives to be obtained with a yield of 73–88% (Figure 9, reaction 9.1). When the reaction was carried out in 1-methoxyethyl-3-methylimidazolium trifluoroacetate [MOEMIM]TFA, the same products were obtained with higher yields. This may be attributed to the ability of [MOEMIM]TFA to hydrogen-bond with aromatic/heterocyclic/1,2-phenylenediamine [54]. Other diaryl-substituted thiazolidinone derivatives have been synthesized in analogous reactions in *N*-methylpyridinium tosylate. In this case, the reactions were carried out for longer and at a higher temperature (3 h at 120 °C), and the products were obtained in yields of 80–93% [55]. In the case where, instead of an amine, an aromatic *o*-diamine was used, the reaction product was thiazolobenzimidazole derivatives (Figure 9, reaction 9.2). Reactions of this type were conducted in [BMIM]BF_4_ and [MOEMIM]TFA, and consistently, the products were obtained with slightly higher yields in [MOEMIM]TFA: 83–94% yields [54].

**Figure 10:**
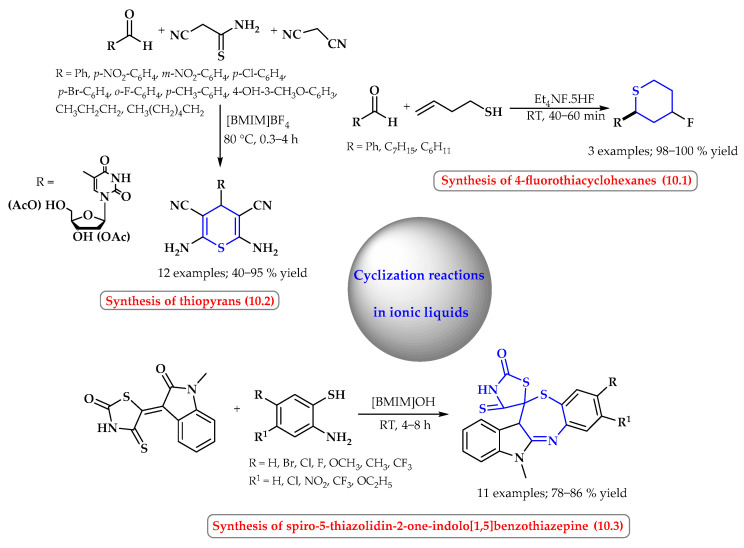
Synthesis of six- and seven-membered *S*-heterocycles in ionic liquids. Heterocyclic fragments of molecules containing sulfur are marked in blue.

Six- and seven-membered *S*-heterocycles are also synthesized in ionic liquids (Figure 10). Kishi et al. reported the highly efficient synthesis of fluorinated thiacyclohexanes via the Prins cyclization of various aldehydes with homoallylic thiols in ionic liquid HF salts (Et_4_NF·5HF) without the use of any organic solvents (Figure 10, reaction 10.1). High yields of up to 96% were obtained selectively more than *cis* products [56].

One-pot multi-component reactions of aldehydes, cyanothioacetamide, and malononitrile promoted via ionic liquid proved to be an efficient approach to the synthesis of thiopyran derivatives (Figure 10, reaction 10.2). In the ionic liquid [BMIM]BF_4_, without the addition of a catalyst, both aromatic and aliphatic aldehydes participated in this reaction smoothly. The methodology proved to be effective in the synthesis of a pyrimidine nucleoside–thiopyran chimera with potential biological activities [57].

Siddiqui et al. performed an efficient method for the synthesis of spiro-5-thiazolidin-2-one-indolo[1,5]benzothiazepine from readily available isatin, 4-thioxothiazolidin-2-one, and 2-aminothiophenol (Figure 10, reaction 10.3). The strategy involves the initial reaction of isatin with 4-thioxo-2-thiazolidin-2-one in [BMIM]OH without using any base, which resulted in the formation of *N*-methyl-3-(2-oxo-4-thioxo-thiazolidinon-5-ylidene)-1,3-dihydroindol-2-one, having α,β-unsaturated (enone) moiety. Then, the product’s Knoevenagel condensation as a result of a thia-Michael addition with 2-aminothiophenol derivatives and intramolecular cyclocondensation gave the final products an excellent yield (78–86%) [58].

**Figure 11:**
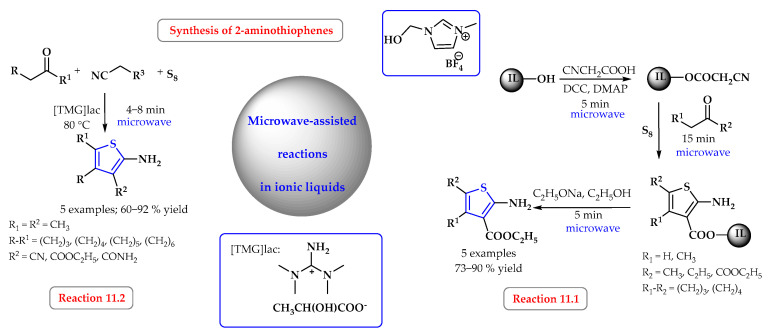
Synthesis of thiazole derivatives in microwave-assisted reactions in ionic liquid. Heterocyclic fragments of molecules containing sulfur are marked in blue.

Ionic liquids are also used as a solvent for radiation-assisted reactions, mainly using microwaves and ultrasounds. The microwave-assisted Gewald synthesis of 2-aminotiophenes was carried out in the ionic liquid 1-(2-hydroxyethyl)-3-methylimidazolium tetrafluoroborate [HYDEMIM]BF_4_ (Figure 11, reaction 11.1). The method used was efficient (73–90%), and the desired products, with a high degree of purity, were obtained without the need for chromatographic purification [59].

Another example of the synthesis of 2-aminotiophenes under the influence of microwaves was the reaction carried out by Chavan et al. in the ionic liquid 1,1,3,3-tetramethylguanidine lactate [TMG][lac] (Figure 11, reaction 11.2). This liquid acts not only as a solvent but also as a catalyst. In the microwave-assisted reaction, the desired products were obtained in a few minutes with a yield of 60–92%, while in the conventional method, a maximum yield of 90% was obtained with heating at 80 °C for several hours. It is also important that the ionic liquid is reusable and can be used in several cycles without a significant loss of catalytic activity [60].

**Figure 12:**
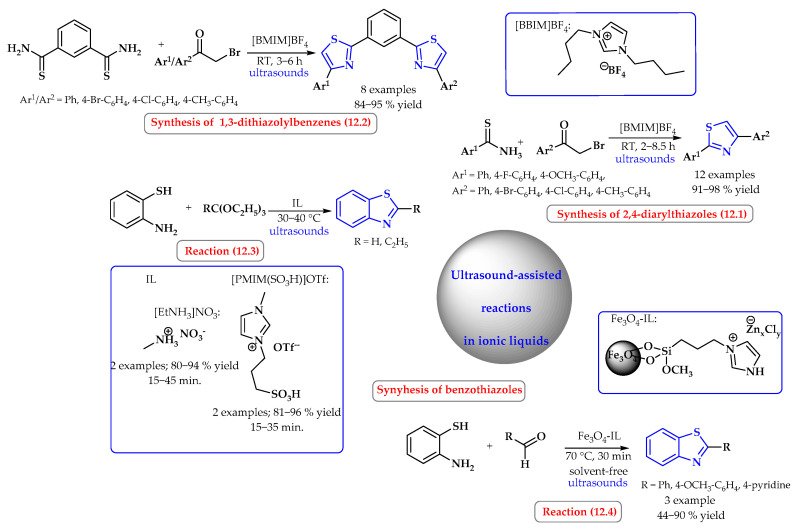
Synthesis of thiazole derivatives in ultrasound-assisted reactions in ionic liquid. Heterocyclic fragments of molecules containing sulfur are marked in blue.

Synthesis of 2,4-diarylthiazoles from arylthioamides and α-bromoacetophenones in [BMIM]BF_4_ were also carried out under ultrasound irradiation at room temperature (Figure 12, reaction 12.1). These compounds were synthesized with excellent yields (91–98%) and some electron deficiency, as well as electron richness, in both arylthioamides and α-bromoacetophenones. Although the differences in the transformation times are slight, generally, the electron-rich arylthioamides were superior to the electron-deficient ones in this regard. Similar results were also observed in the case of substituted α-bromoacetophenones. However, the longest reaction times were observed for the substrates bearing electron-withdrawing groups. When 1,3-benzenedithioamide was used in an analogous reaction, 1,3-dithiazolylbenzenes were obtained with 84–95% yields (Figure 12, reaction 12.2) [61].

Benzothiazoles were obtained from the corresponding thioamines via a reaction with CH(OEt)_3_ and EtC(OEt)_3_ using the readily available, recyclable, Brønsted-acidic ionic liquids ethylammonium nitrate [EtNH_3_]NO_3_ and 1-methyl-3-pentylimidazolium trifluoromethanesulfonate [PMIM(SO_3_H)]OTf under mild conditions with high yields (80–96%) (Figure 12, reaction 12.3). The contents were sonicated in a thermostatted, ultrasonic cleaning bath. Both ionic liquids showed similar activity, although slightly higher yields were observed at shorter or similar times for the reaction taking place in [PMIM(SO_3_H)]OTf [62].

Benzothiazoles were also obtained via cyclization reactions of 2-aminothiophenol and benzaldehyde, 4-methoxybenzaldehyde or 4-pyridinecarboxaldehyde with moderate-to-good yields using imidazolium chlorozincate(II) ionic liquid supported with Fe_3_O_4_ nanoparticles (LAIL@MNP) under solvent-free sonication (Figure 12, reaction 12.4) [63].

**Figure 13:**
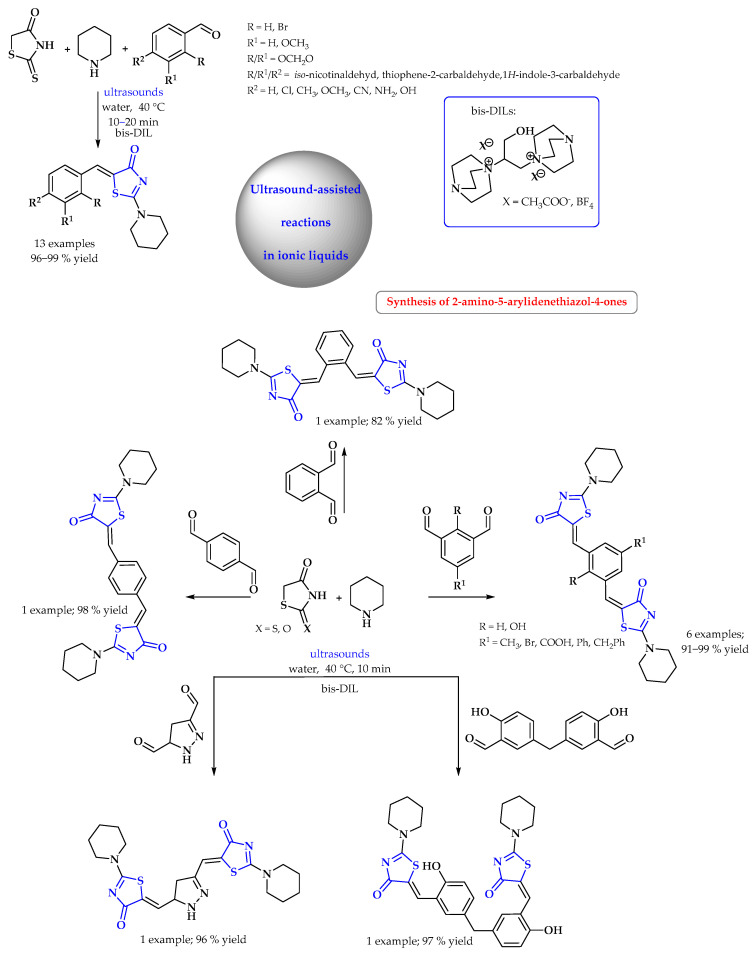
Synthesis of thiazol-4-one derivatives in ultrasound-assisted reactions in ionic liquid. Heterocyclic fragments of molecules containing sulfur are marked in blue.

Arafa and Mourad presented the sonosynthesis of a broad scope of mono- and bis-2-amino-5-arylidenethiazol-4-ones using new DABCO-based ILs under sustainable reaction conditions (Figure 13). The authors investigated the efficiency of bis-DILs (DABCO_2_-C_3_-OH]2X) as powerful ILs in promoting the Knoevenagel condensation reaction of commercially obtainable rhodamine, piperidine, and mono- and dialdehydes. The reaction was carried out “on water” with the addition of an ionic liquid [64].

**Figure 14:**
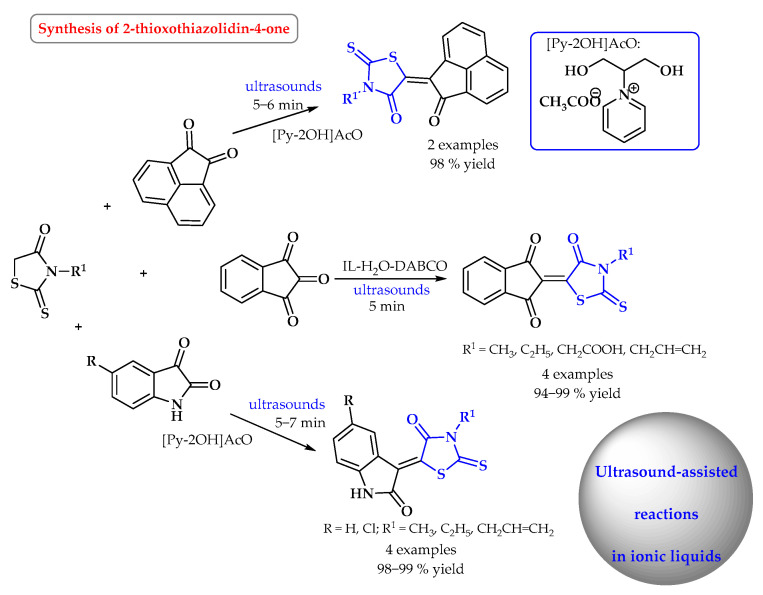
Synthesis of 2-thioxothiazolidin-4-one derivatives in ultrasound-assisted reactions in ionic liquid. Heterocyclic fragments of molecules containing sulfur are marked in blue.

In the synthesis of 2-thioxothiazolidin-4-one derivatives, the ionic liquid ([Py-2OH]OAc) was employed as a promoter in the DABCO-catalyzed Knoevenagel reactions between various cyclic active methylene derivatives and ninhydrin under ultrasonic irradiation (Figure 14). All of the reactions studied went cleanly and smoothly, and the resulting Knoevenagel condensation compounds were recovered in high yields without detecting the aldol intermediates in the end products. The noticeable efficiency of the IL might be attributed to the presence of two hydroxyl groups that acted as a protic additive in promoting the reaction [65].

Many chemical reactions in the presence of organometallic catalysts are also carried out with ionic liquids as solvents. Often, in such reactions, ionic liquids are assigned the role not only of a solvent but also of stabilizing agents for both the metal-active species and intermediates of the catalytic system [66].

**Figure 15:**
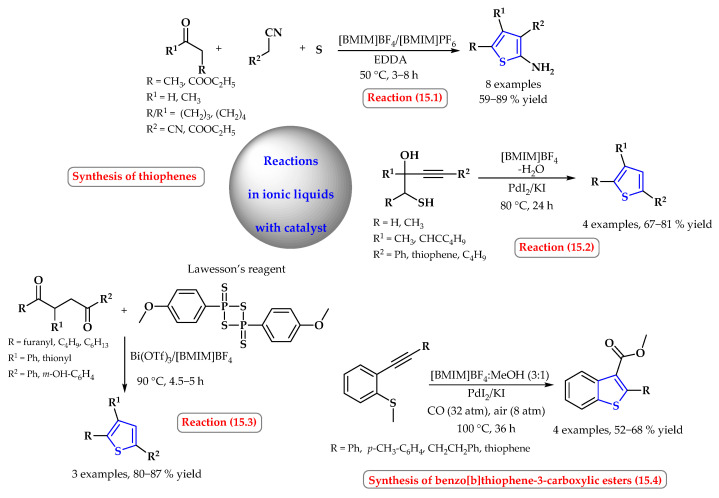
Synthesis of thiophene derivatives using organometallic catalyst in ionic liquids. Heterocyclic fragments of molecules containing sulfur are marked in blue.

2-Aminothiophenes were synthesized in an ionic liquid in the presence of an ethylenediammonium diacetate (EDDA) catalyst in the standard Gewald synthesis (Figure 15, reaction 15.1). Compared to molecular solvents, this method was characterized by better efficiency. In [BMIM]PF_6_ or [BMIM]BF_4_ at 50 °C the process efficiency was 59–89% [67].

In the heterocyclodehydration reaction of 1-mercapto-3-yn-2-ols, under mild conditions using ionic liquids as a solvent ([BMIM]BF_4_/OTf/PF_6_/Cl/NTf_2_), 4-butyl-2,3-dimethylthiophene was obtained with efficiency of 28–82%. The reaction was carried out in the presence of catalytic amounts (1–2%) of PdI_2_ in combination with KI (molar ratio KI:PdI_2_ = 10). The best results were obtained in [BMIM]BF_4_. Other substituted thiophenes were also obtained in this solvent with good yields of 67–81% (Figure 15, reaction 15.2). Unlike conventional organic solvents, the catalyst could be recovered in [BMIM]BF_4_ and reused without losing its catalytic activities [68].

1,4-Dicarbonyl compounds were also successfully used in the synthesis of thiophenes, which, in reaction with Lawesson’s reagent, provided the desired products in high yields (Figure 15, reaction 15.3). The reaction used a reusable catalytic system, Bi(OTf)_3_ immobilized on [BMIM]BF_4_. This procedure offers significant improvements in the reaction rate and yield, and it reduces the likelihood of heterocycle decomposition by avoiding highly acidic reaction conditions [69].

Mancuso et al. presented a palladium-catalyzed carbonylative approach to benzothiophene-3-carboxylic esters, starting from simple and readily available building blocks [2-(methylthio)phenylacetylenes, CO, alcohol, and O_2_ (from air)] (Figure 15, reaction 15.4). In addition to standard organic solvents, the reaction was successfully carried out in the ionic liquid [BMIM]BF_4_. The simple PdI_2_/KI catalytic system obtained the desired products in fair yields (52–68%). An additional advantage of the reaction carried out in an ionic liquid is the possibility of recycling the catalytic system several times without an appreciable loss of activity [70].

**Figure 16:**
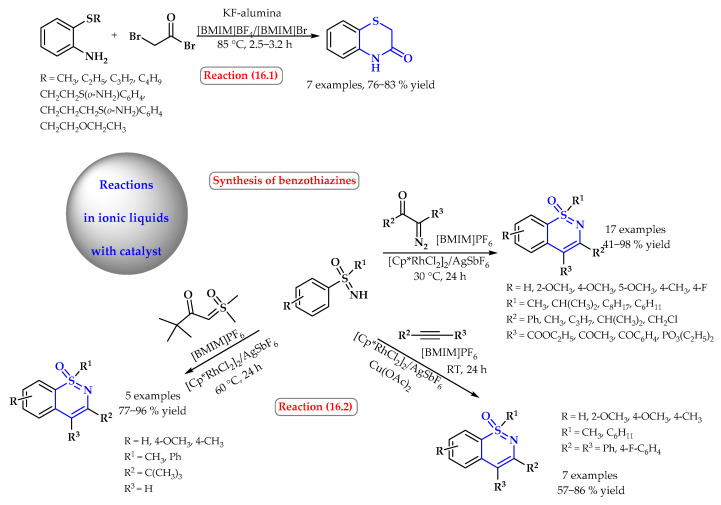
Catalytic synthesis of benzothiazine derivatives in ionic liquids. Heterocyclic fragments of molecules containing sulfur are marked in blue.

However, during the synthesis of 3-oxo-1,4-benzothiazine, the KF-alumina catalytic system in the ionic liquid [BMIM]Br and [BMIM]BF_4_ was used (Figure 16, reaction 16.1). The cyclocondensative transformation of various alkylsulfanylphenylamines with bromoacetyl bromide leads to the corresponding products with yields up to 83%. This procedure shares the advantage of improved yields, the easier separation of the product, a small volume of organic solvent consumption, and, last but not least, the recyclability of the ionic liquid giving an approximately constant yield of the product in consecutive cycles [71].

1,2-benzothiazines have been synthesized in ionic liquids via the Rh(III) catalyzed C-H activation/cyclization of NH-sulfoximines with various coupling partners (diazo compounds, sulfoxonium ylides, and diphenylacetylenes) (Figure 16, reaction 16.2). The best results were obtained using [BMIM]PF_6_ as the solvent. The catalytic system, namely Cp*Rh(III)/AgSbF_6_/[BMIM]PF_6_, still maintained good catalytic activity even after ten cycles. The transformation proceeds with excellent functional group tolerance and high yields [72].

**Figure 17:**
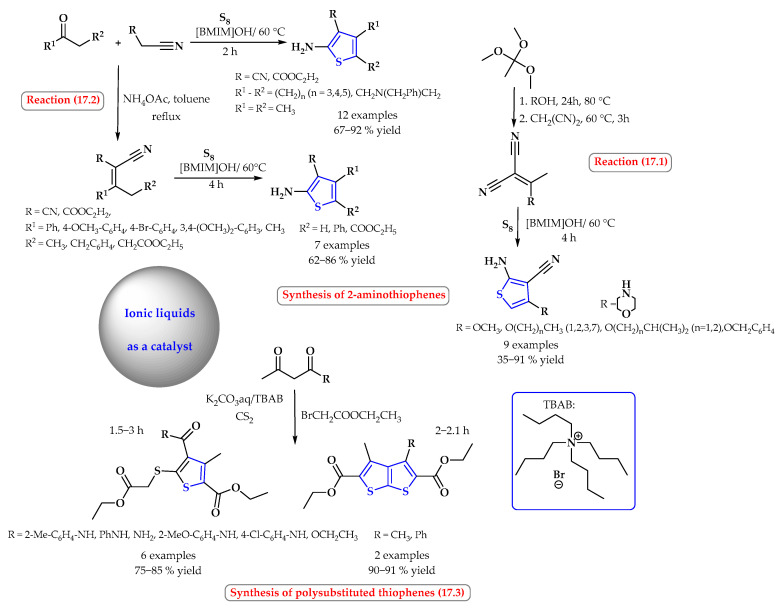
Synthesis of thiophene derivatives using ionic liquid as a catalyst. Heterocyclic fragments of molecules containing sulfur are marked in blue.

In some reactions, the ionic liquid acts as a catalyst. For example, the synthesis of 2-aminothiophenes was carried out in a simple, efficient, and environmentally friendly procedure based on the Gewald reaction using a basic ionic liquid, [BMIM]OH, as both the catalyst and the solvent (Figure 17). At a temperature of 60 °C, in the condensation reaction with elemental sulfur S_8_, 28, a variety of alkyl, aryl, alkoxy, and alkylamino-2-aminothiophenes in good yields (35–91%) were obtained after filtration (Figure 17, reaction 17.1). Thus, 4,5-dialkyl-2-aminothiophenes were obtained as a result of direct condensation of ketones, activated nitrile, and sulfur, while 4-aryl-2-amino/2,3,4,5-tetrasubstituted thiophenes and 4-alkoxy/alkylamino-2-aminothiophenes were obtained via the cyclization of ylides with sulfur (Figure 17, reaction 17.2). The reaction was carried out for 2 to 4 h [73].

Wang et al. reported a facile and convenient one-step synthesis of polysubstituted thiophenes and polysubstituted thieno[2,3-*b*]thiophenes from 1,3-dicarbonyl compounds via a tetrabutylammonium bromide-catalyzed TBAB reaction (Figure 17, reaction 17.3). The reaction took place in water in the presence of potassium carbonate. The products were obtained with high yields of up to 91%. However, the ionic liquid was recycled after the reaction products were recovered [74].

**Figure 18:**
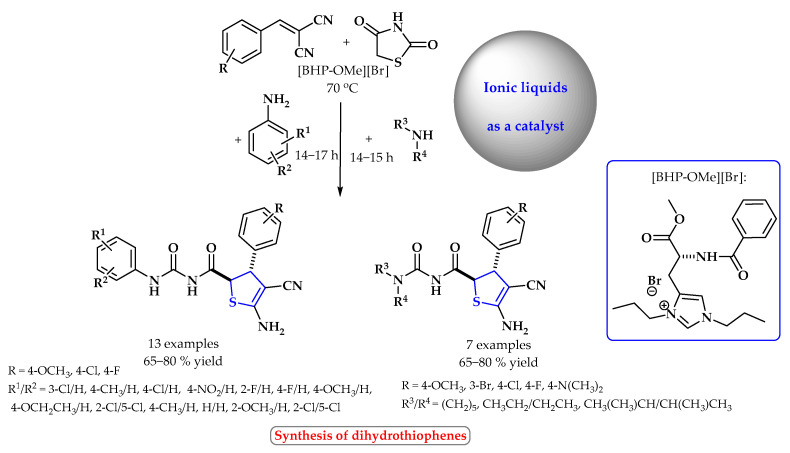
Synthesis of dihydrothiophene derivatives using ionic liquid as a catalyst. Heterocyclic fragments of molecules containing sulfur are marked in blue.

Kumar et al. developed a three-component, one-pot methodology in which 2-arylidenemalononitrile, 1,3-thiazolidinedione, and aliphatic/aromatic amines were added to the amino acid-based ionic liquid [BHPOMe]Br and water to form dihydrothiophenes (Figure 18). The functional ionic liquid [BHP-OMe]Br was found to catalyze the synthesis of diastereoselective dihydrothiophenes and also tacrine derivatives. Diastereomerically pure dihydrothiophene derivatives were obtained with a yield of up to 80% [75].

**Figure 19:**
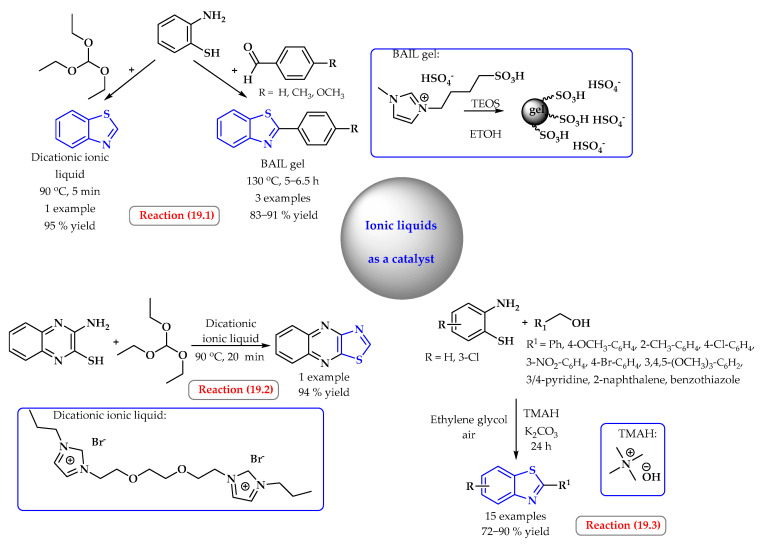
Synthesis of benzothiazole derivatives using ionic liquid as a catalyst. Heterocyclic fragments of molecules containing sulfur are marked in blue.

Nguyen et al. synthesized benzothiazoles in a reusable Brønsted acidic ionic liquid gel (BAIL gel) obtained by treating 1-methyl-3-(4-sulfobutyl)-1*H*-imidazolium hydrogen sulfate with tetraethyl orthosilicate (TEOS) (Figure 19, reaction 19.1). The BAIL gel represents a new catalyst for the synthesis of benzothiazoles from 2-aminothiophenol and aromatic aldehydes. This method can be used in industry; it is characterized by high efficiency, it is easy to process, it is neutral to the atmosphere, and the catalyst can be recycled [76].

A dicationic ionic liquid based on imidazolium cation was designed, synthesized, and successfully used as a catalyst for the one-pot synthesis of benzothiazoles from 2-aminothiophenol, *ortho*-phenylenediamines, and triethyl orthoformate (Figure 19, reaction 19.2). The ethyleneoxy bridge present in the ionic liquid additionally increases the solubility of organic compounds. Reaction products were obtained in a simple and convenient procedure with high conversion and in a short time with the possibility of reusing the catalyst [77].

Badhani et al. presented the tetramethylammonium hydroxide (TMAH)-catalyzed oxidative coupling of *ortho*-phenylene diamines and 2-aminobenzenethiols for the synthesis of benzothiazoles under metal-free conditions by utilizing oxygen from air as the terminal oxidant (Figure 19, reaction 19.3). Substituted 2-aminobenzenethiols and other alcohols such as trimethoxy benzyl alcohol, pyridin-3-ylmethanol, pyridin-4-ylmethanol, furan-2-ylmethanol, and naphthalen-2-ylmethanol were applied, and they provided the desired products in good yields (72–90%) [78]. 

**Figure 20:**
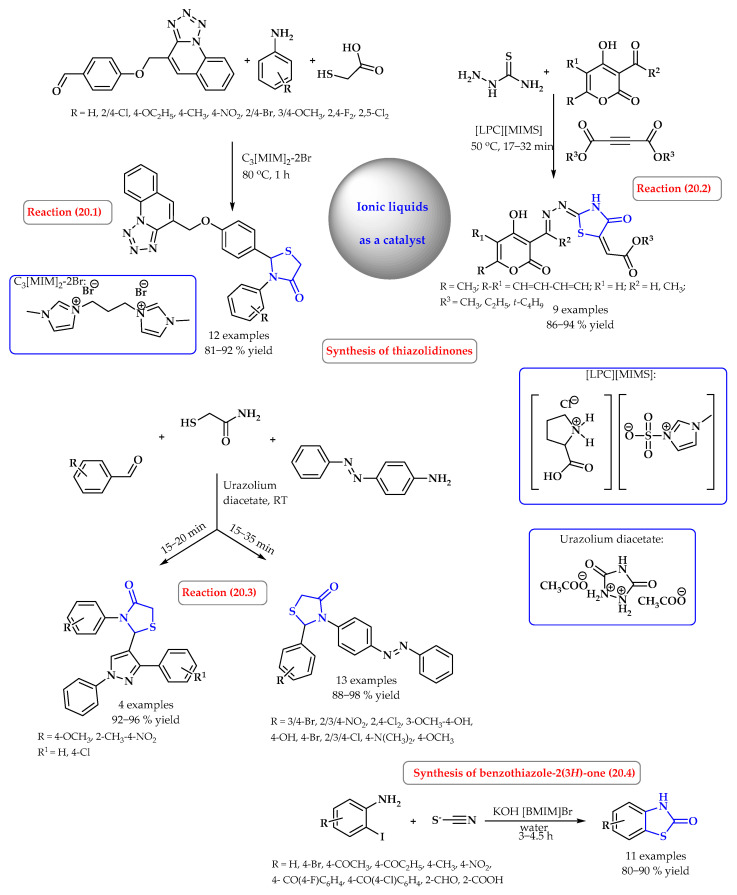
Synthesis of benzothiazolone and thiazolidinone derivatives using ionic liquid as a catalyst. Heterocyclic fragments of molecules containing sulfur are marked in blue.

Dicationic ionic liquid, (3-methyl-1-[3-(methyl-1*H*-imidazolium-1-yl)propyl]-1*H*-imidazolium dibromide (C_3_[MIM]_2_–2Br), binary ionic liquid mixture L-prolinium chloride/1-methylimidazolium-3-sulfonate ([LPC][MIMS]), and the Brønsted acid ionic liquid urazolium diacetate were also used as catalysts in the synthesis of thiazolidinone derivatives. 3-substituted phenyl-2-(4-(tetrazolo[1,5-a]quinolin-4-ylmethoxy)phenyl)thiazolidin-4-ones were prepared in dicationic ionic liquid (C_3_[MIM]_2_–2Br) as a medium and the catalyst by performing the one-pot cyclocondensation of 4-(tetrazolo[1,5-a]quinolin-4-ylmethoxy)benzaldehyde, anilines, and mercaptoacetic acid (Figure 20, reaction 20.1) [79]. On the other hand, hydrazono-4-thiazolidinones were obtained under the catalysis of a natural-based binary ionic liquid in a one-pot reaction of thiosemicarbazide and 4-hydroxylpyran-2*H*-ones (Figure 20, reaction 20.2) [80]. 1,3-thiazolidine-4-ones (azo dispersive dyes family) were synthesized via a multicomponent reaction of various aldehydes, thioglycolic acid, and 4-aminoazobenzene using the Brønsted acid ionic liquid urazolium diacetate as a catalyst (Figure 20, reaction 20.3) [81].

Meanwhile, Waseem et al. presented an expeditious, facile, ionic liquid-catalyzed, water-accelerated synthesis of substituted benzothiazole-2(3*H*)-one derivatives from readily available 2-iodoaniline and potassium thiocyanate under basic conditions (Figure 20, reaction 20.4). Products were obtained at an excellent yield of up to 90%. The strategy involved nucleophilic substitution forming N-C and S-C bonds, resulting in the desired heterocyclic scaffold [82].

**Figure 21:**
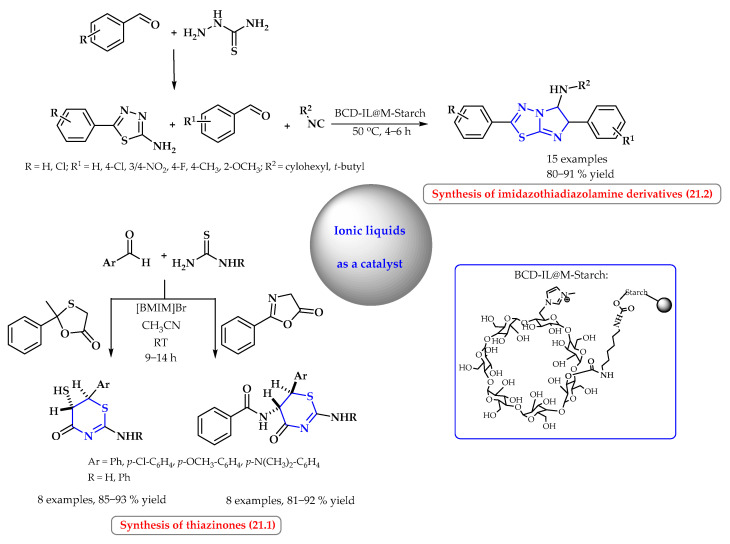
Synthesis of imidazothiadiazolamine and thiazinone derivatives using ionic liquid as a catalyst. Heterocyclic fragments of molecules containing sulfur are marked in blue.

Yadav et al. developed an original and practical method for the synthesis of potentially pharmaceutically important thiazinone derivatives from readily available simple substrates (Figure 21, reaction 21.1). The synthesis is highly diastereoselective and involves tandem Knoevenagel, Michael, and ring-transformation reactions in a one-pot procedure. *Trans*-selectivities were observed using [BMIM]Br ionic liquid as an environmentally friendly catalyst. The expected heterocycles were formed in very high yields at room temperature using acetonitrile as the solvent [83].

Bahadorikhalili at al. used the catalytic properties of the ionic liquid-β-cyclodextrin, which was anchored to magnetic starch (denoted βCD-IL@M-Starch) in the synthesis of imidazo[2,1-*b*][1,3,4]thiadiazol-5-amine and imidazo[1,2-a]pyridin-3-amine derivatives (Figure 21, reaction 21.2). The βCD-IL@M-Starch catalyst showed very good activity in the synthesis of the desired products from the corresponding benzaldehyde, semicarbazide, benzaldehydes, and isocyanides. The catalyst was shown to be magnetically reusable, and it achieved very good results in 10 sequential reactions [84].

## 5. Synthesis of *S*-Heterocycles in Deep Eutectic Solvents as Green Solvents

Deep eutectic solvents (DESs) are now widely acknowledged as a new class of ionic liquid (IL) analogs because they share many characteristics and properties with ILs. The difference is that DESs are systems formed from a eutectic mixture of Lewis or Brønsted acids and bases, which can contain a variety of anionic and/or cationic species, while ILs are formed from systems composed primarily of one type of discrete anion and cation [85]. 

**Figure 22:**
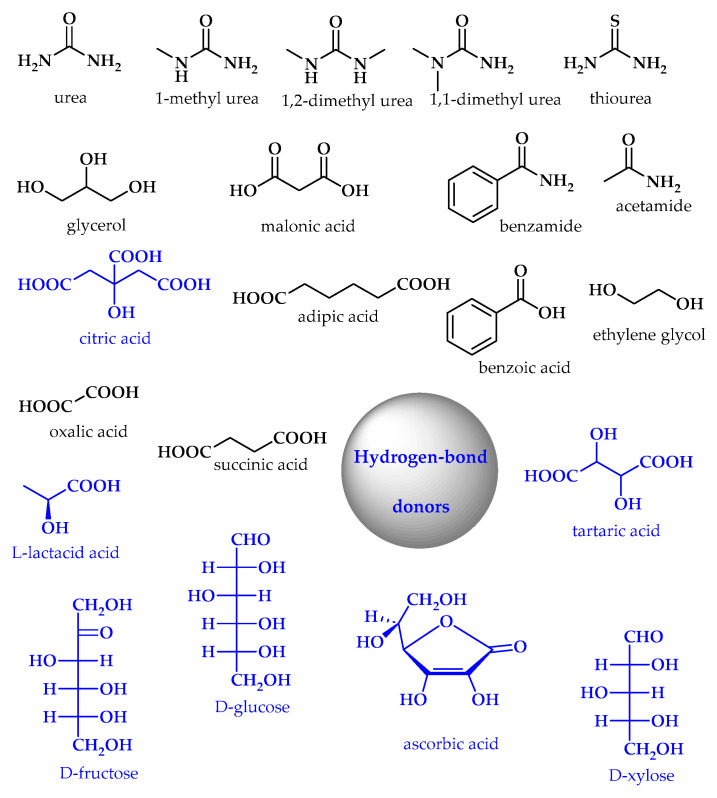
Examples of hydrogen-bond donors (NDES (natural deep eutectic solvents) ingredients are marked in blue).

**Figure 23:**
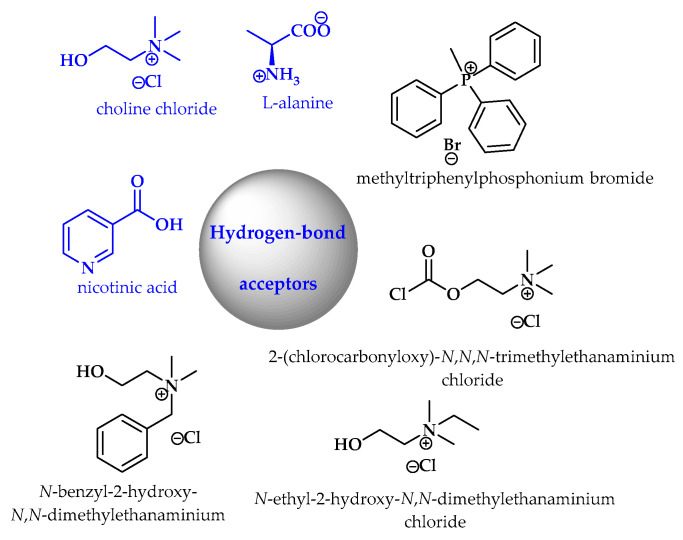
Examples of hydrogen-bond acceptors (NDES ingredients are marked in blue).

Different types of DESs have been described, and most contain hydrogen-bond acceptors (HBAs) and hydrogen-bond donors (HBDs) (Figure 22 and Figure 23). Alternatively, they may also contain metal salts or hydrated metal salts [12]. 

The melting points of DESs are lower than those of the individual components, and they are often prepared by mixing two solid reagents to form a liquid product. Usually, they are obtained via the complexation of a quaternary ammonium salt with a metal salt or hydrogen bond donor (HBD). The charge delocalization occurring through hydrogen bonding between, for example, a halide ion and the hydrogen-donor moiety is responsible for the decrease in the melting point of the mixture relative to the melting points of the individual components [12,85].

Deep eutectic solvents can be described according to the general formula Cat ^+^ X ^−^ zY, where Cat ^+^ is an ammonium, phosphonium, or sulfonium cation, and X is a Lewis base, generally a halide anion. The complex anionic species are formed between X ^−^ and either a Lewis or Brønsted acid Y (*z* is a number of Y molecules that interact with the anion) [85]. 

DES components often come from renewable sources (e.g., choline chloride (ChCl), urea, glycerol (Gly), lactic acid, carbohydrates, polyalcohols, amino acids, and vitamins). Therefore, their biodegradability is extraordinarily high, and their toxicity is non-existent or very low. Given their minimal ecological footprint, the cheapness of their constituents, the tunability of their physico-chemical properties, and their ease of preparation, DESs are successfully and progressively replacing often hazardous and volatile organic compounds (VOCs) in many fields of science [86]. DESs composed entirely of plant metabolites (such as ammonium salts, sugars, and organic acids) are labeled natural deep eutectic solvents (NADESs).

DESs have been proposed as environmentally benign alternative solvents for synthesis. However, the possibility of using DESs as solvents for synthesis has not received as much focused research attention as utilizing DESs as alternative solvents for metal finishing applications [85]. Many organic reactions are catalyzed by acids or bases, and these components appear in DESs. Thus, a DES can be used not only as a reaction medium but also as a catalytic active species for some reactions, and in some cases, it can also represent part of the starting materials [87]. 

**Figure 24:**
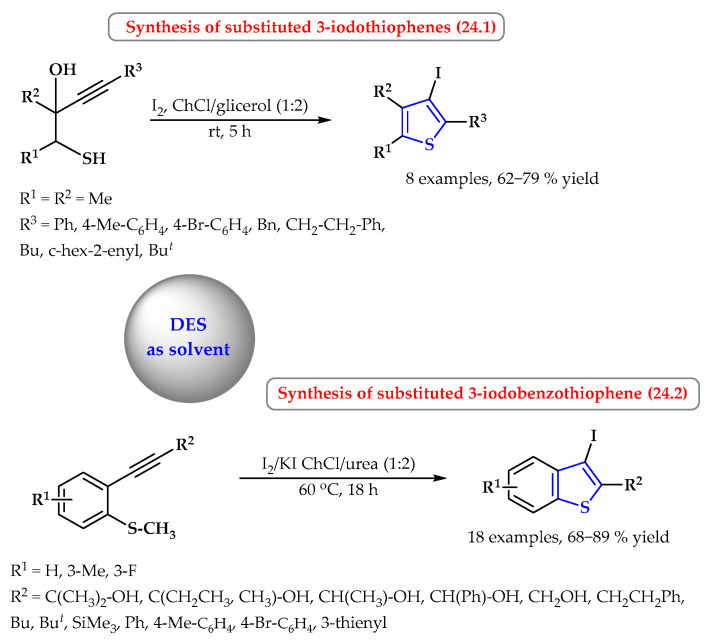
Synthesis of thiophene derivatives in DESs as solvents. Heterocyclic fragments of molecules containing sulfur are marked in blue.

Mancuso et al. presented the application of green eutectics in the synthesis of thiophene derivatives (Figure 24) [88]. The *S*-heterocyclization and iodocyclization of readily available 1-mercapto-3-yn-2-ols were carried out in the choline chloride/glycerol system (ChCl/Gly mixed in a molar ratio of 1:2) as a non-conventional green solvent at room temperature (Figure 24, reaction 24.1). Eight 3-iodothiophene derivatives substituted with alkyl or aryl groups were obtained in good yields (62–79%).

However, the iodocyclization reaction of 2-methylthiophenylacetylenes to derivatives of benzothiophenes takes place using choline chloride and urea mixed in a molar ratio of 1:2 (Figure 24, reaction 24.2) [89]. The reaction proceeds most efficiently in the presence of I_2_/KI at a temperature of 60 °C; increasing the temperature negatively affects the yield of the synthesis. The process successfully afforded a variety of other substituted derivatives containing the iodobenzothiazole system. A total of 26 derivatives were obtained with yields of 62–89%. 

**Figure 25:**
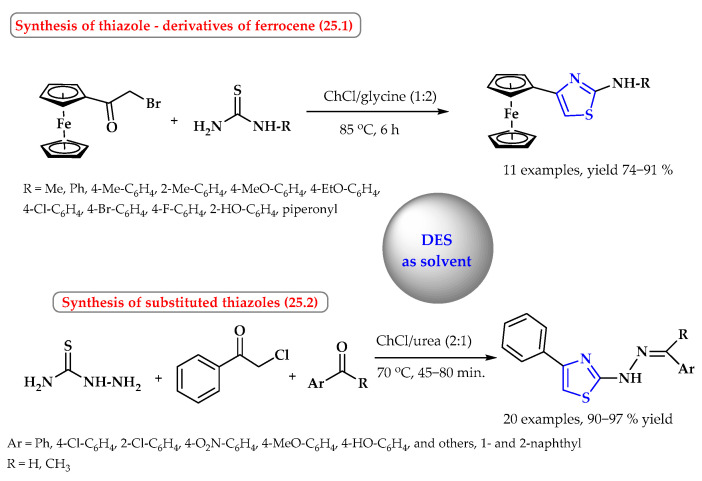
Synthesis of thiazole derivatives in DESs as solvents. Heterocyclic fragments of molecules containing sulfur are marked in blue.

An interesting example of the use of EDS is the *S*-cyclization reaction of α-bromoacetylferrocene with substituted thioamides in the choline chloride/glycine system (1:2) at 85 °C (Figure 25, reaction 25.1). This is an example of the green Hantzsch reaction. The synthetic strategy has attractive features, such as mild and environmentally benign reaction conditions, the possibility of recycling, the simplicity of the experiment, and an easy work-up procedure. Under optimized conditions, substituted thiazoles were obtained in high yields from 74 to 91% [90].

The one-pot synthesis of hydrazinyl-4-phenyl-1,3-thiazole derivatives was also carried out in EDS, consisting of choline chloride and urea (ChCl/urea mixed at a molar ratio of 2:1). The derivatives were obtained by reacting phenacyl chloride with thiosemicarbazide and aryl aldehydes or ketones (Figure 25, reaction 25.2) [91]. The products formed excellent yields of 90–97% over short reaction times (45–80 min) under environmentally friendly conditions.

**Figure 26:**
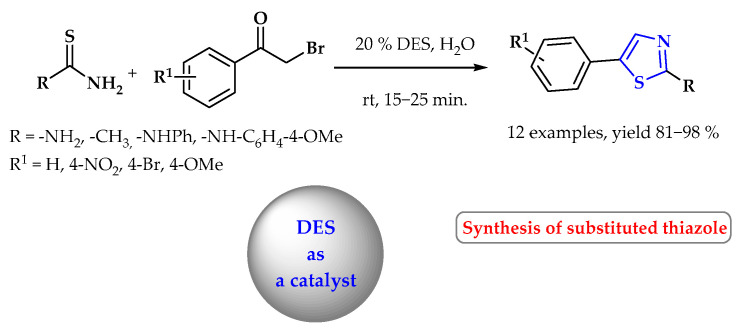
Synthesis of 2-methyl- and 2-aminothiazole derivatives using DES as catalyst. Heterocyclic fragments of molecules containing sulfur are marked in blue.

Deep eutectic solvents have also been used as catalysts. A simple deep eutectic ammonium catalyst, easily synthesized from choline chloride and urea, catalyzed the synthesis of 2-methyl- and 2-aminotiazole derivatives in the reaction of substituted phenacyl bromides with thiourea or thioacetamide derivatives. The use of DES as a catalyst allowed final products to be obtained in a short time (15–25 min) with high yields (81–98%) (Figure 26) [92].

## 6. Synthesis of *S*-Heterocycles in Glycerol as a Green Solvent

Glycerol is a polyalcohol that has been utilized in many different fields, such as the pharmaceutical and food industries, tobacco, and cellulose films. The sustainability and low cost of glycerol make it a good green solvent compared to other organic solvents which are hazardous, volatile, toxic, and harmful compounds. Despite the fact that glycerol is a solvent and is selected for many reactions, there are some limits to its use. Glycerol is highly viscous; therefore, it should be fluidified with a co-solvent, or the reactions can proceed at temperatures higher than 60 °C because then its viscosity is much lower. Due to the presence of 3 hydroxyl groups, which can be mentioned as acidic sites in the molecule, glycerol may be involved in the reaction. Glycerol has enough length and a donor atom in which it can obtain complexes with metal catalysts, resulting in unwanted side products and/or the unreactivity of catalysis. Despite these limitations, glycerol is willingly used as a green solvent in organic synthesis [18]. 

**Figure 27:**
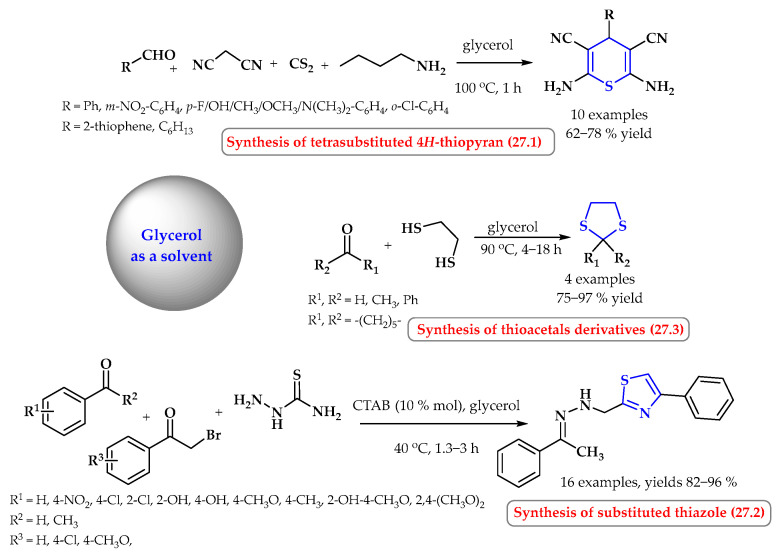
Synthesis of *S*-heterocycles in glycerol. Heterocyclic fragments of molecules containing sulfur are marked in blue.

Environmentally friendly methods for the synthesis of thiopyran in the work of Mitra et al. consisted of a one-pot reaction of the appropriate aldehyde, malononitrile, carbon disulfide, and 1-butylamine (Figure 27, reaction 27.1) [93]. The advantage of the developed method is that is has cheap reagents and no need to use a catalyst. In the standard procedure, the process was carried out in glycerol for 1 h at a temperature of 100 °C. Under these conditions, 10 aromatic, heterocyclic and aliphatic thiopyran derivatives were obtained with yields ranging from 62 to 78%. The authors showed that the reaction carried out in glycerin is more efficient than that in ethylene glycol. The use of ethylene glycol as a solvent (12 h, 100 °C) resulted in obtaining only trace amounts of the product, and conducting the reaction in glycerine under the same conditions ensures the reaction with 80% efficiency [93].

Tiwari et al. presented a convenient, eco-friendly, one-pot, multi-component synthesis for obtaining 2,4-disubstituted thiazoles from substituted carbonyl compounds, phenacyl bromides, and thiosemicarbazide (Figure 27, reaction 27.2) [94]. The reactions were carried out in glycerin with 10% cetyl trimethyl ammonium bromide (CTAB). The use of micellar catalysis in glycerol was the key aspect of this methodology, which possesses superiority compared to glycerol alone. The 16 thiazole derivatives were obtained with yields of 82–96%. The use of benzaldehyde in the reaction with unsubstituted phenacyl bromide for 2 h provided a final product with a yield of 92%, while the use of acetophenone instead of benzaldehyde in an analogous reaction (2 h) resulted in a slight deterioration of the reaction yield (85%). Substituted benzaldehydes (4-Cl, 4-NO_2_, 4-OCH_3_, and 2-OH) provided high yields in these reactions (up to 96% at a time of approx. 1.3–2 h). However, a slight decrease in efficiency and an increase in the reaction time for activating substituents were observed (84–86%, time 2.5–3 h). In the case of reactions with substituted acetophenones and substituted phenacyl bromides, the reaction times were 2–2.5 h, and the yields were 82–90%.

Glycerin was used as an efficient and recyclable solvent in the thioacetalization reaction of aldehydes and ketones. A reaction with 1,2-ethanedithiol in glycerol at 90 °C gave the corresponding cyclic thioacetals a good yield (75–97%) (Figure 27, reaction 27.3) [95].

**Figure 28:**
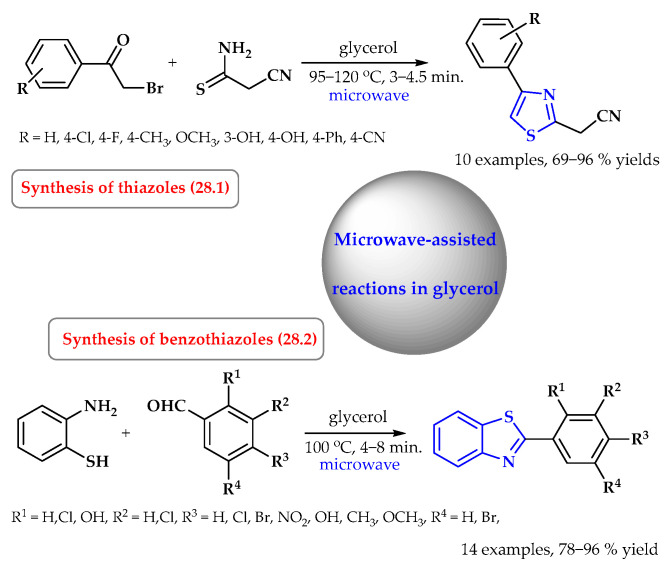
The microwave-assisted reactions of the synthesis of *S*-heterocycles in glycerol. Heterocyclic fragments of molecules containing sulfur are marked in blue.

Glycerol is also readily used as a solvent for microwave-assisted reactions (Figure 28). The synthesis of thiazole derivatives is an example of such a reaction. The substituted 2-cyanomethyl-4-phenylthiazoles were synthesized in a reaction of cyanoacetamide and 2-bromoacetophenones with substituents in a phenyl ring under focused microwave irradiation using glycerol as a solvent (Figure 28, reaction 28.1). The method allows for the synthesis of products with excellent yields of up to 96% and short reaction times (a few minutes), and the work-up is easy [96]. The synthesis of benzothiazoles via the condensation of 2-aminothiophenol with aldehydes under CEM-focused microwave irradiation conditions with glycerol as a solvent and without any catalyst was also carried out (Figure 28, reaction 28.2). The use of a variety of aromatic aldehydes bearing electron-withdrawing substituents, such as chloro, bromo, and nitro groups, afforded high yields for the products. In addition, a hydroxy group can be tolerated under these conditions, and good yields of the desired products were obtained. A reaction of heterocyclic aldehydes also provided 2-heterocyclic-substituted benzothiazoles with good yields [97]. 

## 7. Synthesis of *S*-Heterocycles in Ethylene Glycol as a Green Solvent

Ethylene glycol (EG) is the simplest diol, and it possesses two hydroxyl groups in the molecule. Ethylene glycol is an inexpensive, low-toxicity, relatively non-volatile, and hygroscopic solvent. EG is odorless, miscible with polar solvents (water and alcohols), and slightly soluble in non-polar solvents (benzene and chloroform). Ethylene glycol is produced from ethylene oxide, and it can be also produced from renewable biomass [98]. EG has many applications in chemistry (in protecting the carbonyl group, antifreeze applications, the synthesis of polyethylene terephthalate, and others). In green chemistry, it can be an interesting and promising solvent for various organic transformations [99].

**Figure 29:**
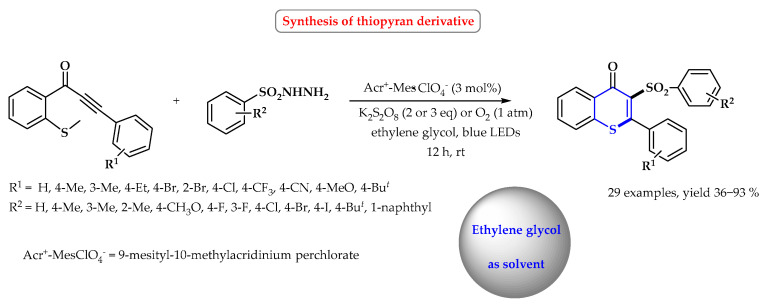
Synthesis of *S*-heterocycles in ethylene glycol. Heterocyclic fragments of molecules containing sulfur are marked in blue.

Jiang et al. performed photocatalytic cascade sulfonation/cyclization reactions with ethylene glycol as a green solvent at room temperature [98]. The reaction used benzenesulfonyl hydrazide and methylothiolated alkynone. The synthesis of sulfone-containing heterocycles was carried out in the presence of various oxidants (K_2_S_2_O_8_, BPO, and O_2_), and the catalyst was 9-mesityl-10-methylacridinium perchlorate (Arc^+^-Mes·ClO_4_^−^). The cascade sulfonation/cyclization required the use of the irradiation of blue light (λ = 460 nm). Under optimized conditions, a series of 29 substituted sulfonated thioflavones were obtained with yields from 36 to 93% (Figure 29).

## 8. Synthesis of *S*-Heterocycles in Polyethylene Glycol (PEG) as a Green Solvent

Polyethylene glycol (PEG) is the polymeric form of ethylene glycol; the number designated in PEG indicates the average molecular weight. PEG is available in a variety of molecular weights (PEG-600, PEG-200, and others). Poly(ethylene glycol) (PEG) is a new class of green, biocompatible reaction media. PEG is a polymer that is highly soluble in water, it is recyclable, and it is a stable solvent. PEG also possesses unique properties: a low cost, low inflammability, reduced toxicity, and high miscibility with organic compounds. PEG can be used as not only a solvent but also as a catalyst. When it is used as a solvent, one of its advantages in reactions allows for the creation of smaller amounts of by-products in the reactions taking place. When acting as a catalyst, it can be an alternative to a phase-transfer catalyst (PTC), replacing more expensive and less environmentally friendly catalysts. Moreover, PEG has been used in industrial chemistry (shampoo production, biodiesel production, and many others) [100].

**Figure 30:**
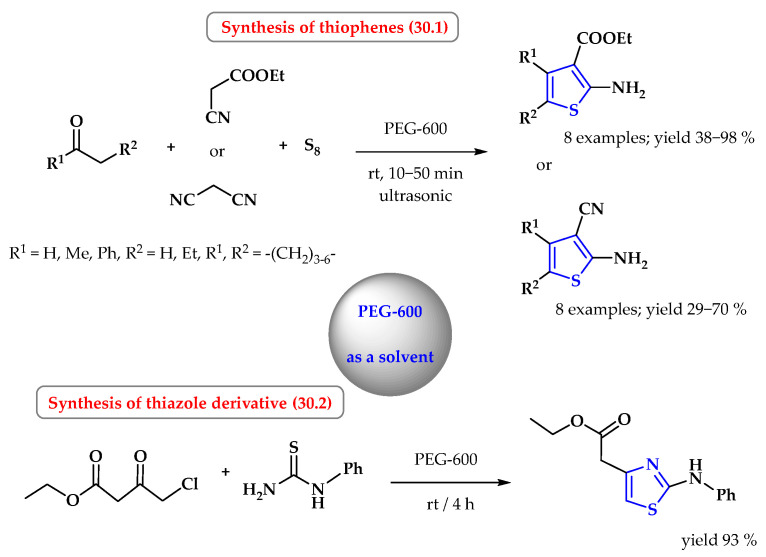
Synthesis of thiophene and thiazole derivatives in PEG-600. Heterocyclic fragments of molecules containing sulfur are marked in blue.

One of the applications of PEG may be the use of PEG in the Gewald reaction. Abdarzadeh et al. presented an efficient method of synthesizing functionalized 2-aminotiophene derivatives in a one-pot, three-component Gewald reaction from enolizable carbonyl compounds, malononitrile (or ethyl cyanoacetate), and elemental sulfur using PEG 600 as the reaction medium (Figure 30, reaction 30.1). Reactions were carried out under mild conditions without the use of a basic-catalyst simple-processing procedure and with the possibility of recycling the reaction medium. The best results were obtained by carrying out the reaction at room temperature using ultrasonic irradiation. In a short time, from 10 min to a maximum of 50 min, 16 thiophene derivatives were obtained with yields ranging from 29 to 98% [101]. 

The one-pot, three-component synthesis of the substituted thiazoles can be prepared using PEG-600 as a solvent. Ethyl 4-chloroacetoacetate with phenylthiourea with equimolar reagents in PEG-600 undergoes cyclization within 4 h to the substituted thiazole system at room temperature with an efficiency of 93% (Figure 30, reaction 30.2) [102]. For comparison, the reaction taking place in glycerine at a temperature of 100 °C requires 5 h, and the product is obtained with a lower yield of 77%. The same reaction in ethylene glycol at 100 °C takes place with an efficiency of 76% in 5.5 h. Interestingly, the process using PEG-600 at a temperature of 100 °C and for 3.5 h results in the formation of the final thiazole with a slightly lower efficiency than at room temperature (90%).

**Figure 31:**
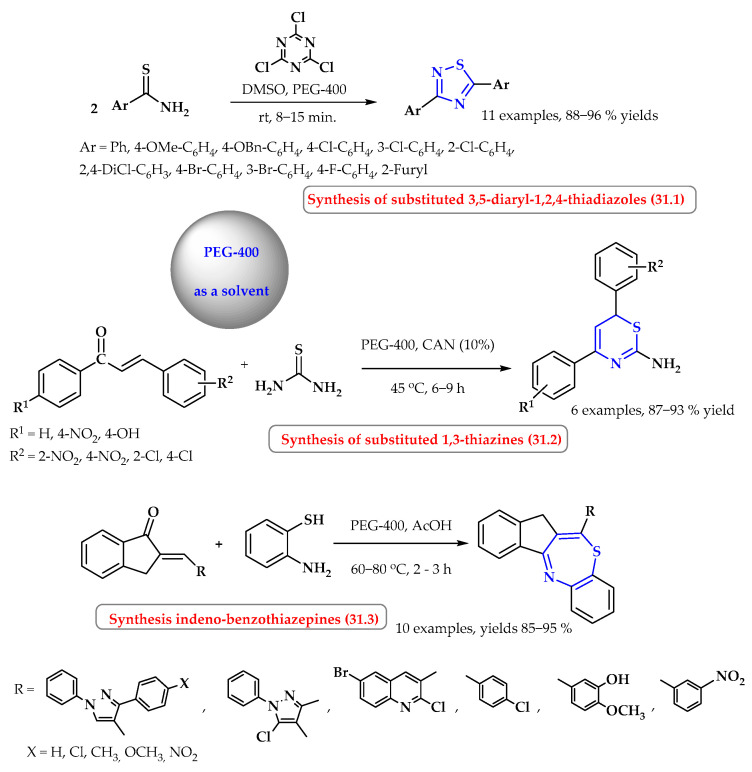
Synthesis of *S*-heterocycles in PEG-400. Heterocyclic fragments of molecules containing sulfur are marked in blue.

The synthesis of derivatives of 1,2,4-thiadiazoles using 2,4,6-trichloro-1,3,5-triazine and the appropriate thioamides was carried out in PEG-400 at room temperature for 11 different thioamides, and the final products were obtained in a short time (8–15 min) with high efficiency (88–96%). The reaction occurs via intramolecular oxidative dimerization and the cyclization of substituted thioamides (Figure 31, reaction 31.1) [103].

Singh et al. performed a simple, inexpensive, and efficient one-pot, multicomponent synthesis of 1,3-thiazine in PEG-400 catalyzed by cerium ammonium nitrate (CAN, 10 mol%). 1,3-Thiazine derivatives were synthesized using acetophenones, aromatic aldehydes, and thiourea (Figure 31, reaction 31.2) [104,105]. The yield and reaction time were influenced by substituents in the aromatic ring of the starting substrates. For unsubstituted acetophenone, benzaldehyde, and thiourea, the reaction occurred with a yield of 95% in 7 h (10%mol CAN). The presence of an electron-withdrawing substituent (-NO_2_ group) slightly reduced the product yield (87–89%) with the time extended to 9 h. In turn, the introduction of an electron-releasing group (-OH) shortened the reaction time and increased the efficiency of the reaction (93%, 6 h). Finally, six distinguished 1,3-thiazines were obtained with yields of 87–93% [104].

Acharya et al. presented the synthesis of indenobenzothiazepines derivatives using PEG-400 as a solvent (Figure 31, reaction 31.3) [106]. The reaction was carried out at a temperature of 60–80 °C for 2–3 h. The substrates were 1-indanone derivatives and 2-aminothiophenol. Under the above conditions, a series of 10 indeno-benzothiazepines were obtained with very high yields of 85–95% [106].

**Figure 32:**
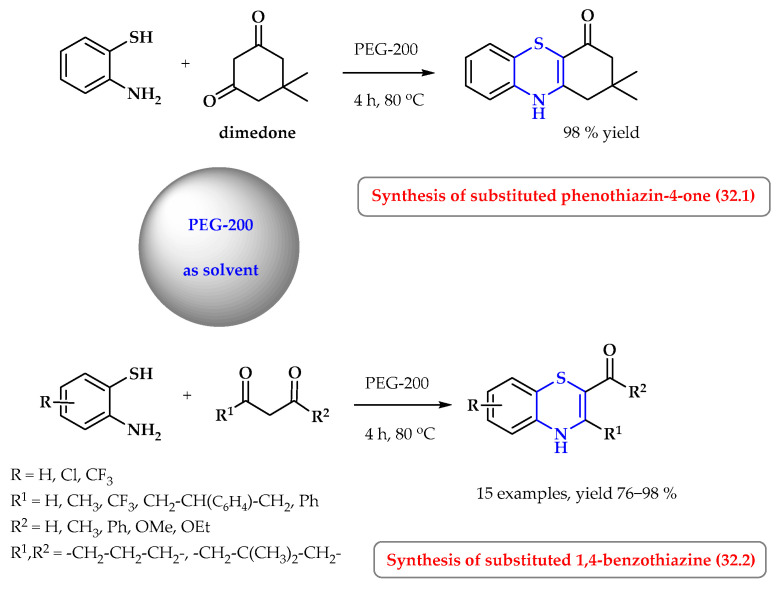
Synthesis of thiazine derivatives in PEG-200. Heterocyclic fragments of molecules containing sulfur are marked in blue.

A mixture of dimedone and 2-aminophenol was treated in different solvents and temperatures (Figure 32, reaction 32.1) [107]. Among various solvents, the best results were achieved with PEG-200 for 4 h at 80 °C. Extending the running time and increasing the temperature did not increase the reaction efficiency (98%). The same reaction, carried out at room temperature (PEG-200, rt, 24 h), did not produce the expected product. By comparison, the reaction carried out in glycerin as a solvent for 5 h and at a temperature of 90 °C allowed the product to be obtained with a yield of 60%. Trace amounts of the final product were obtained by conducting the process in methanol and ethanol. The cyclization reactions of 2-aminobenzenethiol with 1,3-dicarbonyl compounds were performed under optimized conditions (PEG-200 for 4 h at 80 °C), resulting in 15 substituted 1,4-benzothiazines. The reaction yields were also very high (76–98%) (Figure 32, reaction 32.2).

## 9. Synthesis of *S*-Heterocycles in Sabinene as a Green Solvent

**Figure 33:**
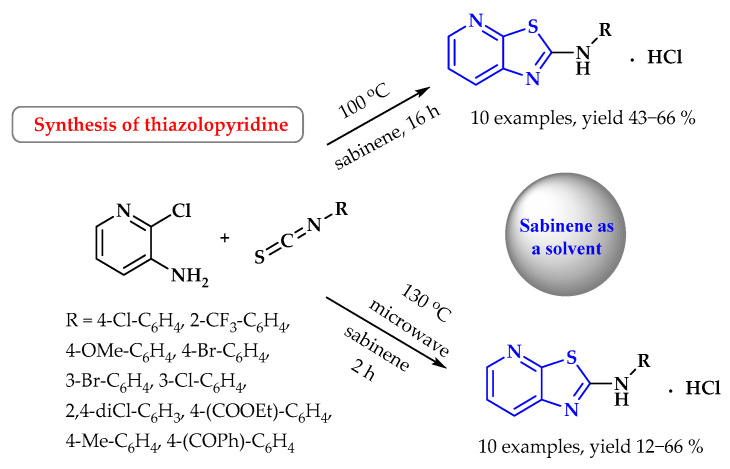
Synthesis of thiazolopyridine derivatives using sabinene as a solvent. Heterocyclic fragments of molecules containing sulfur are marked in blue.

Sabinene (4-methylidene-1-(propan-2-yl)bicyclo[3.1.0]hexane) is an organic compound belonging to monoterpenes, an unsaturated hydrocarbon with the general formula C_10_H_16_. It is non-toxic and can be recycled through distillation. It is used, among other uses, in the perfume industry. It occurs naturally in juniper, marjoram, holm oak, black pepper, and others [108]. It can be a green solvent for the synthesis of various thiazolo[5,4-*b*]pyridines via thermal activation or microwave irradiation. After the reaction conditions were optimized for the synthesis of thiazolo[5,4-*b*]pyridines derivatives in sabinene, it was found that it is most effective at 100 °C for 16 h or at 130 °C for 2 h in a microwave-assisted reaction. The desired products were obtained from the appropriate isothiocyanates and 3-amino-2-chloropyridine under thermal or microwave activation with yields of 43–66% and 12–66%, respectively (Figure 33) [108].

## 10. Conclusions

In conclusion, we have presented a review of the methods of synthesis of various *S*-heterocyclic compounds containing five-, six-, and seven-membered heterocyclic compounds containing a sulfur atom or atoms, as well as those with other heteroatoms and fused ring systems using green solvents such as water, ionic liquids, deep eutectic solvents, glycerol, ethylene glycol, polyethylene glycol, and sabinene. It has been shown that the use of green solvents determines the attractiveness of conditions for many reactions; for others, this use constitutes a real compromise between efficiency and mild reaction conditions.
